# Population genetic structure of *Indoplanorbis exustus* (Gastropoda: Planorbidae) in Thailand and its infection with trematode cercariae

**DOI:** 10.1371/journal.pone.0297761

**Published:** 2024-01-26

**Authors:** Abdulhakam Dumidae, Chanakan Subkrasae, Jiranun Ardpairin, Supawan Pansri, Raxsina Polseela, Aunchalee Thanwisai, Apichat Vitta

**Affiliations:** 1 Department of Microbiology and Parasitology, Faculty of Medical Science, Naresuan University, Phitsanulok, Thailand; 2 Centre of Excellence in Medical Biotechnology (CEMB), Faculty of Medical Science, Naresuan University, Phitsanulok, Thailand; 3 Center of Excellence for Biodiversity, Faculty of Sciences, Naresuan University, Phitsanulok, Thailand; Niigata University of Pharmacy and Medical and Life Sciences, JAPAN

## Abstract

*Indoplanorbis exustus* is a freshwater gastropod belonging to the family Planorbidae. This snail is widely distributed across the tropics and plays an important role as the intermediate host for trematodes. However, relatively little is understood regarding the genetic relationship between *I*. *exustus* and trematodes. The goals of this study were to investigate the current transmission status of trematode cercariae in *I*. *exustus* in Thailand and to examine the genetic diversity, genetic structure, and demographic history of *I*. *exustus*. We collected 575 *I*. *exustus* from 21 provinces across six regions of Thailand and investigated cercarial infections by using the shedding method. *I*. *exustus* from two provinces were infected with cercarial trematodes, and two types of cercarial stages were molecularly identified as furcocercous cercaria and xiphidiocercariae. Phylogenetic tree analysis based on 28S rDNA and ITS2 sequences demonstrated that furcocercous cercaria and xiphidiocercariae were closely clustered with a clade of *Euclinostomum* sp. and *Xiphidiocercariae* sp., respectively. Phylogenetic and network analyses of *I*. *exustus* haplotypes based on the COI, 16S rDNA, and ITS1 genes demonstrated four main clades. Only snails in clade A were distributed in all regions of Thailand and harbored trematode cercariae. The level of genetic diversity of *I*. *exustus* was relatively high, but most populations were not genetically different, thus suggesting the appearance of gene flow within the *I*. *exustus* populations. Overall, the haplotype network was star-shaped, thus suggesting the recent demographic expansion of populations. This result was also supported by the unimodal mode of the mismatch distribution graph and the large negative values of the neutrality tests. Therefore, the *I*. *exustus* snail was likely another freshwater snail of the invasive species in Thailand. This information will aid in monitoring the spread of the parasitic trematodes carried by *I*. *exustus* from different populations.

## Introduction

*Indoplanorbis exustus* (Deshayes, 1834) (Gastropoda: Planorbidae) is a freshwater snail found in various tropical regions. Currently, *I*. *exustus* is widely distributed in Asia, Africa, and the Caribbean region [1–4]. This organism has been reported as an intermediate host for various trematode parasites (Trematoda: Digenea) in veterinary and medical fields [5, 6]. *I*. *exustus* is known as the intermediate host for *Schistosoma indicum*, *S*. *nasale*, and *S*. *spindale*, as well as other trematodes such as *Clinostomum giganticum* and *Echinostoma* spp. [5, 6]. Moreover, *I*. *exustus* has been reported in association with outbreaks of cercarial dermatitis in humans in Thailand, Malaysia, Laos, and India [6–9]. Cercarial dermatitis, also known as swimmers itch is caused by an acute allergic reaction in the skin that is a result of repeated penetration of the skin by larvae (cercariae) of animal blood flukes. Humans become infected with trematode cercariae by contact water in ponds, rice paddy fields, and lakes contaminated with cercariae, which are shed by infected snails into freshwater [10].

In addition, *I*. *exustus* is an important intermediate host for parasitic trematodes in veterinary and medical settings. This snail is commonly found attached to aquatic plants in small ponds, pools, lakes, and rice paddy fields, including in semi-permanent pool flooding fields. *I*. *exustus* acts as an important plant pest because it reduces the vigor of some crops by killing seeds or seedlings, destroying stems or growing points, or causing leaf damage, which subsequently causes economic loss [11, 12].

Within the snail population, derived data on genetic variation can provide valuable information about colonization and spread into new areas [13]. The most commonly used genes for genetic analysis in *I*. *exustus* snails are the mitochondrial, cytochrome c oxidase subunit I (COI), and 16S rDNA genes [1, 3, 4]. Additionally, the internal transcribed spacer I (ITS1) region, nuclear 18S rRNA, and 28S rRNA genes have been used as genetic markers for studying phylogeny [14]. The phylogenetic analysis of *I*. *exustus* using mitochondrial DNA sequences has demonstrated four to five genetically distinct clades. These clades were always congruent, thus suggesting that *I*. *exustus* was a species complex [3–5]. Moreover, it was initially divided into the clades found in northern India and Cratonic India, as well as being ubiquitous in Thailand, Nepal, the Philippines, Sundaic in Indonesia, and Malay-Occidental and Arabian clades [1]. Subsequently, these were classified into five clades (specifically, clades A to E), of which clade E was widely distributed, encompassing South Asia, Southeast Asia, and Southwest Asia [15]. Recently, Saijuntha et al. (2021) [4] reported the genetic variation of *I*. *exustus* from new geographical areas in South and Southeast Asia, which phylogenetic tree revealed five major clades (A to E). Clade A, clade B, and clade C were found only in South Asia, whereas clade D was specifically found in Southeast Asia. The high self‐fertilization capacity of *I*. *exustus* likely facilitates geographical expansion [16] and may have important parasitological implications, as it is the natural intermediate host of digenean trematodes. Certainly, when the vector of parasitosis is present in a geographical area, no factor excludes the emergence of the disease if the parasite is introduced.

Given that *I*. *exustus* may represent a species complex, it is also necessary to consider host-parasite relationships. Devkota et al. (2015) [5] first proposed the existence of a snail-host complex and classified *I*. *exustus* into four different lineages. Lineage I included *I*. *exustus* infected with echinostome and *Schistosoma* sp. Lineage II comprised snails infected with xiphidiocercariae, strigeids, and sanguinicolid cercariae. Lineage III consisted of snails positive for *S*. *nasale*, and lineage IV comprised snails infected with strigeids, sanguinicolids, xiphidiocercariae, *S*. *indicum*, *S*. *spindale*, *S*. *nasale*, and *Schistosoma* sp. This study could not identify any specific host lineage-parasite species relationships based on parasitological screening. This suggests that schistosomes may be able to switch hosts within the *Indoplanorbis* species complex. This confirms the importance of an enlarged parasitological survey, despite the absence of host specificity for *Indoplanorbis*.

In invasive host populations, the number of available genetic variants allows them to ‘fight’ the infection. The level-standing genetic variation of invasive populations may be crucial in successfully adapting to new environments and in resisting diseases [17]. However, relatively little is understood regarding the genetic relationship of hosts/parasites between *I*. *exustus* and digenean trematodes in Thailand. Thus, this study aimed to investigate the current status of trematode cercariae in *I*. *exustus* in Thailand and to analyze the genetic diversity of *I*. *exustus* by using the mitochondrial cytochrome c oxidase subunit I (COI), 16S rDNA, and nuclear internal transcribed spacer I region (ITS1).

## Materials and methods

### Ethical approval

The experiments involved with the use of invertebrate animals (snails) in this study were approved by the Center for Animal Research of Naresuan University (Project Ethics Approval No: NU-AQ640803). The experimental protocols of biosafety and biosecurity were approved by the Naresuan University Institutional Biosafety Committee (Project Approval No: NUIBC MI 64-09-34).

### Collection and morphological identification of snails

*Indoplanorbis exustus* snails were randomly collected from 21 provinces in six geographical regions of Thailand (Table 1). The snails were collected from their natural habitats, such as paddy fields, irrigation canals, ponds, wetland ponds, and lotus ponds, by using handpicking and scooping methods. The snails were kept in a plastic box with water and air ventilation and were transported to the Department of Microbiology and Parasitology, Faculty of Medical Science, Naresuan University, Phitsanulok, Thailand. All of the snails were cleaned with several changes of tap water and were preliminarily identified through the comparison of shell morphology, as described by Brandt [18] and Frandsen [19]. Morphological characteristics of *I*. *exustus* were taxonomically identified based on a discoid shell with brownish or olive colors, dorso-ventrally flat shapes with rapidly increasing whorls, and dimensions nearly as high as wide [18, 19].

**Table 1:** Collection data of *I*. *exustus* in this study.

Collection site	Code	Latitude/Longitude	Geographical region	Habitat	No. of *I*. *exustus* collected (cercaria positive)
Ban Kaeng, Tron District, Uttaradit Province	UTT1	17.4582/100.1674	North	Paddy field	1 (0)
Tha Sop Sao, Mae Tha District, Lamphun Province	LPN1	18.4382/99.0969	North	Irrigation canal	9 (0)
Mae Tha, Mae Tha District, Lampang Province	LPG1	18.1725/99.5615	North	Paddy field	1 (0)
Thung Lui Lai, Khon San District, Chaiyaphum Province	CPM2	16.6007/101.7428	Northeast	Wetland pond	17 (0)
Na Fai, Phu Phaman District, Khon Kaen Province	KKN2	16.7320/101.8440	Northeast	Wetland pond	2 (0)
Kut Chap, Kut Chap District, Udon Thani Province	UDN2	17.3901/102.4995	Northeast	Wetland pond	1 (0)
Nong Wua So, Nong Wua So District, Udon Thani Province	UDN3	17.1837/102.4293	Northeast	Wetland pond	6 (0)
Tha Pho, Mueang Phitsanulok District, Phitsanulok Province	PLK1	16.7118/100.1977	Central	Pond	19 (0)
	PLK6	16.6926/100.2249		Paddy field	9 (0)
	PLK9	16.7191/100.1879		Paddy field	4 (0)
	PLK10	16.7090/100.2034		Paddy field	23 (0)
	PLK11	16.6824/100.2303		Paddy field	3 (0)
Khlong Maphlap, Si Nakhon District, Sukhothai Province	STI1	17.3623/99.9892	Central	Lotus pond	3 (0)
Ban Na, Wachirabarami District, Phichit Province	PCT1	16.5127/100.1519	Central	Paddy field	3 (0)
Sam Ngam, Sam Ngam District, Phichit Province	PCT2	16.5287/100.2189	Central	Paddy field	4 (0)
Pho Sai Ngam, Bueng Na Rang District, Phichit Province	PCT3	16.1187/100.1266	Central	Lotus pond	14 (0)
Nam Ko, Lom Sak, District, Phetchabun Province	PNB3	16.7769/101.1922	Central	Lotus pond	5 (0)
Tha Chanuan, Manorom District, Chai Nat Province	CNT1	15.3717/100.1546	Central	Paddy field	18 (0)
	CNT2	15.3413/100.1677		Paddy field	14 (0)
Hang Nam Sakhon, Manorom District, Chai Nat Province	CNT3	15.3111/100.1842	Central	Irrigation canal	22 (0)
Chi Nam Rai, In Buri District, Sing Buri Province	SBR1	15.0609/100.3247	Central	Paddy field	31 (0)
	SBR2	15.0764/100.3115		Irrigation canal	7 (0)
Namtan, In Buri District, Sing Buri Province	SBR5	14.9625/100.3717	Central	Paddy field	41 (0)
Nam Song, Phayuha Khiri District, Nakhon Sawan Province	NSN1	15.4261/100.1180	Central	Irrigation canal	83 (1)
Nong Krot, Banphot Phisai District, Nakhon Sawan Province	NSN2	16.0211/100.1140	Central	Paddy field	2 (0)
Chorakhe Rong, Chaiyo District, Ang Thong Province	ATG1	14.6493/100.4764	Central	Paddy field	3 (0)
Ban Len, Bang Pa-in District, Ayuthaya Province	AYA1	14.2282/100.6114	Central	Paddy field	1 (0)
Thonglang, Ban Na District, Nakhon Nayok Province	NYK1	14.1862/101.0387	Central	Paddy field	2 (0)
Nam Ruem, Mueang Tak District, Tak Province	TAK1	16.8900/99.2211	West	Irrigation canal	15 (0)
Wang Prachop, Muang Tak District, Tak Province	TAK5	16.9145/99.3335	West	Paddy field	3 (0)
Phlio, Laem Sing District, Chanthaburi Province	CTI1	12.5146/102.1597	East	Lotus pond	1 (0)
Saen Suk, Mueang Chon Buri District, Chon Buri Province	CBI1	13.2803/100.9268	East	Lotus pond	74 (0)
Sai Khao, Khok Pho District, Pattani Province	PTN2	6.6784/101.0842	South	Paddy field	4 (0)
Kho Hong, Hat Yai District, Songkhla Province	SKA1	7.0113/100.4987	South	Lotus pond	89 (0)
Phawong, Mueang Songkhla District, Songkhla Province	SKA2	7.1542/100.5762	South	Lotus pond	41 (1)
Total					575 (2)

### Screening of infected snails and morphological identification of cercariae

Each individual snail was investigated for cercarial infections by using the shedding method [20]. Snails were individually placed into 50 ml capacity containers (4 cm in diameter and 6.5 cm in height) containing 20 ml of dechlorinated tap water. The container was maintained for 24 h at room temperature [21]. Afterwards, snails were screened for shedding cercariae by using a stereomicroscope. Living cercariae were classified according to morphological characteristics by using cercarial keys according to Chontananarth and Wongsawad (2013) [22], Anucherngchai et al. (2016, 2017) [23, 24], and Dunghungzin and Chontananarth (2020) [25]. After preliminary identification, cercariae from individual snails were collected by using a micropipette under a stereomicroscope, transferred to a 1.5 ml microcentrifuge, and kept at -20°C for DNA extraction. In addition, the body of the snail was removed from its shell, and a small piece of foot tissue (approximately 25 mg) was cut from each individual and stored at -20°C until DNA extraction.

### DNA extraction

Genomic DNA from individual snails and from cercariae was extracted by using the NucleoSpin® Tissue Kit (Macherey-Nagel, Duren, Germany) according to the manufacturer’s instructions. The quality of the genomic DNA was verified on a 0.8% agarose gel in 1× TBE buffer at 100 V for 35 min. Furthermore, the genomic DNA solution was stored at -20°C for later use in the PCR analysis.

### Polymerase chain reaction (PCR) and sequencing

Partial fragments of the mitochondrial COI and 16S rDNA genes and ITS1 of *I*. *exustus* were amplified via PCR by using specific primer pairs. For cercariae, PCR was used to amplify the selected nucleotide regions (28S rDNA for furcocercous cercariae and ITS2 for xiphidiocercariae). The specific primers that were used in the present study are listed in Table 2. All of the polymerase chain reactions (PCRs) were performed by using a Biometra TOne Thermal Cycler (Analytik Jena AG, Jena, Germany). The PCR components (30 μl final reaction volume) contained 15 μl of Quick Taq™ HS DyeMix (Toyobo, Shanghai Biotech, China), 1.5 μl of each primer at 5 μM (0.25 μM), 9 μl of distilled water, and 3 μl of the DNA template (20–200 ng). The details of the PCR parameters that were used in each targeted nucleotide region are shown in Table 2. The amplified products were analyzed by using 1.2% agarose gel electrophoresis at 100 V. The PCR products were then purified by using a NucleoSpin® Gel and PCR Clean-Up Kit (Macherey-Nagel, Germany) according to the manufacturer’s instructions. The purified PCR products were shipped to Macrogen Inc., Seoul, Korea, for sequencing in both forward and reverse directions.

**Table 2:** PCR primers and parameters used in this study.

Gene or region	Primer/(Reference)	Amplicon size (bp)	Cycling conditions	Target organism
COI	LCO1490_forward 5’-GGTCAACAAATCATAAAGATATTGG-3’ HCO2198_reverse 5’-TAAACTTCAGGGTGACCAAAAAATCA-3’ [26]	710	95°C/5 min; 95°C/30 sec, 48°C/30 sec, 72°C/30 sec, 34 cycles; 72°C/10 min	*I*. *exustus*
16S rDNA	16Sar_forward 5’-CGCCTGTTTATCAAAAACAT-3’ 16Sbr_reverse 5’-CCGGTCTGAACTCAGATCACGT-3’ [27]	500		
ITS1	ITS1-S_forward 5’-CCATGAACGAGGAATTCCCAG-3’ 5.8S-AS_reverse 5’-TTAGCAAACCGACCCTCAGAC-3’ [28]	802	94°C/10 min; 94°C/30 sec, 53°C/1 min, 72°C/1 min, 25 cycles; 72°C/7 min	
28S rDNA	CF1_forward 5′-GAGTTGAACTGCAAGCTCTGG-3′ CR2_reverse 5′-TTCGCCCCTATACTCACGTTAT-3′ [29]	877	94°C/5 min; 94°C/30 sec, 50°C/1 min, 72°C/1 min, 35 cycles; 72°C/10 min	Furcocercous cercaria
ITS2	ITS3_forward 5′-GCATCGATGAAGAACGCAGC-3′ ITS4_reverse 5′-TCCTCCGCTTATTGATATGC-3′ [30]	480	94°C/5 min; 94°C/1 min, 56°C/1 min, 72°C/30 sec, 35 cycles; 72°C/10 min	Xiphidiocercaria

### Sequence analysis

The nucleotide sequences were checked for errors and manually edited in the software program SeqMan II (DNASTAR, Madison, WI, USA). All of the nucleotide sequences were aligned by using Clustal W in the MEGA Version 7.0 program [31]. Species identification of *I*. *exustus* and cercariae was supported by a BLASTN search to identify similarities to sequences deposited in the NCBI database (http://blast.ncbi.nlm.nih.gov/Blast.cgi).

### Phylogenetic analysis

Phylogenetic trees were constructed via the maximum likelihood (ML), neighbor-joining (NJ), and Bayesian inference (BI) methods. The ML tree was performed based on the Tamura-Nei model [32], and the NJ tree was analyzed with the Kimura two-parameter model [33] with 1,000 bootstrap replicates by using MEGA Version 7.0 [31]. The BI analysis was performed by using the MrBayes 3.2.0 program [34]. The Bayesian posterior probabilities (BPPs) were estimated by using Markov chain Monte Carlo (MCMC) analysis with 2,000,000 generations. Moreover, trees were sampled every 1,000th generation, thus resulting in 2,000 trees. Although three methods were used for building phylogeny, only the ML tree is shown in the present study. The bootstrap values from the two methods (ML and NJ) and the percentage of Bayesian posterior probabilities are indicated on the branch of the ML tree.

### Network analysis

The number of haplotypes and polymorphic sites were calculated by using DnaSP version 5 [35] and ARLEQUIN version 3.5.1.2 [36]. The haplotype relationships of each COI, 16S rDNA, and ITS1 gene and the combined mtDNA of *I*. *exustus* were estimated by using the median-joining algorithm [37] in Network 5.0.1.1 (**http://www.fluxus-engineering.com**).

### Population analysis

Sequences were analyzed by grouping into two datasets based on which previously published GenBank sequences were added to our dataset, in order to analyze the genetic population. Group 1 included all of the sequences of our *I*. *exustus* population from Thailand (n = 486) complemented with 120 published *I*. *exustus* sequences from other countries, and Group 2 consisted of all of the sequences of our *I*. *exustus* population from different provinces of Thailand (Table 3).

**Table 3:** Number of nucleotide sequences of *I*. *exustus* used for genetic analyses.

Research	COI	16S rDNA	ITS1	Total
Present study	162	162	162	486
Liu et al., 2010 [1]	11	11	-	22
Devkota et al., 2015 [5]	14	14	12	40
Mouahid et al., 2018 [3]	19	19	20	58
Total	206	206	194	606

Haplotype diversity and nucleotide diversity for *I*. *exustus* were calculated in ARLEQUIN version 3.5.1.2 [36]. The genetic differentiation among the populations was calculated in ARLEQUIN based on pairwise F_ST_.

Mismatch distribution analysis was used to test the history of population expansion. The sum-of-squares deviation (SSD) and Harpending’s raggedness index [38] were used to test the deviation from the sudden expansion model [39]. Furthermore, historical demographic expansions were determined by using neutrality tests conducted via the following two approaches: Fu’s *Fs* test [40] and Tajima’s *D* [41] tests, which are related to natural selection. Mismatch distribution analysis and neutrality tests were estimated by using ARLEQUIN.

## Results

### Infection of *I*. *exustus* with trematode cercaria

**Fig 1:**
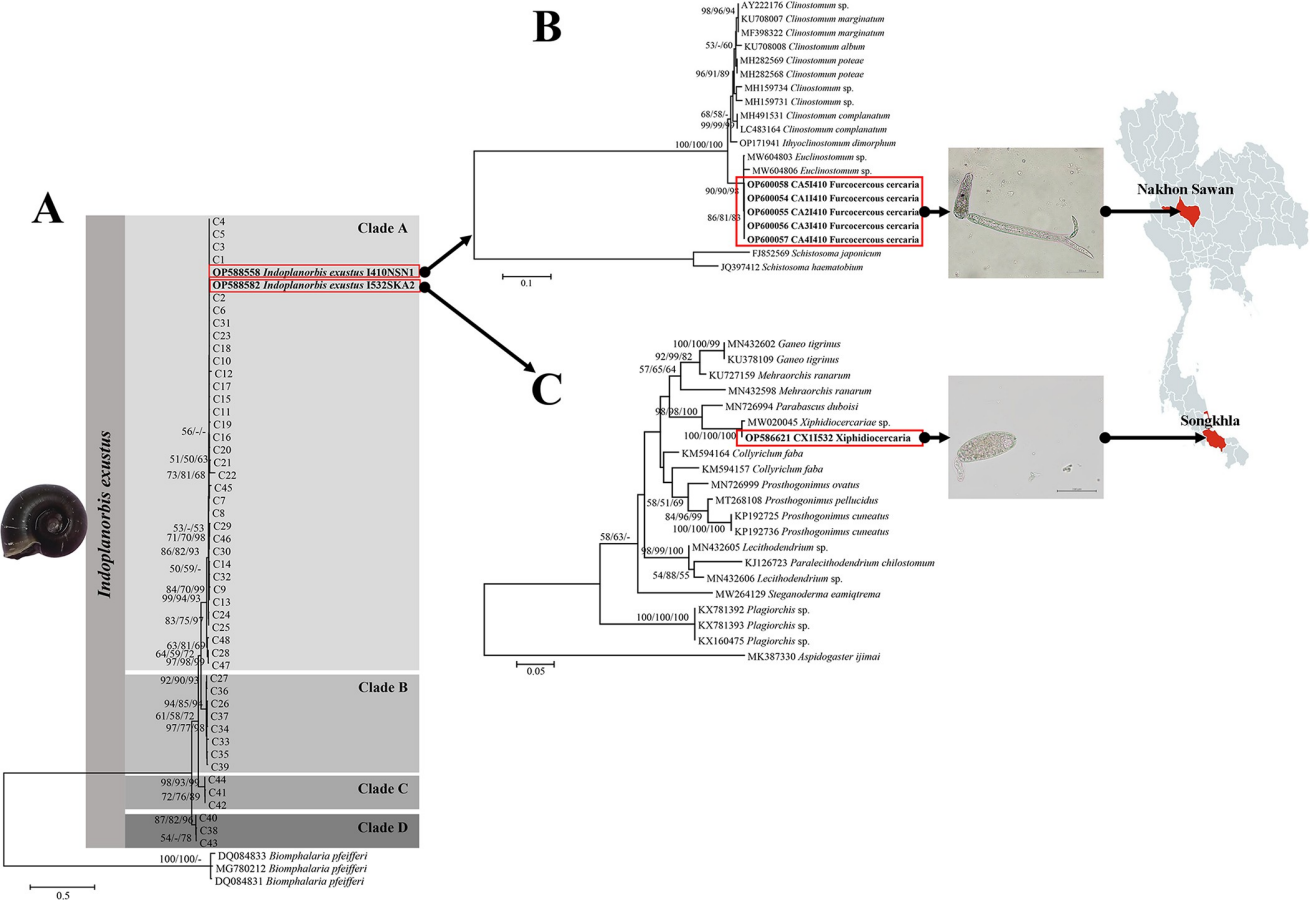
Maximum likelihood phylogenetic tree of *I*. *exustus* and trematode cercariae. (A) ML phylogenetic tree of the haplotypes generated from 206 sequences of a partial COI sequence (569 bp) of *I*. *exustus* (162 sequences from Thailand and 44 sequences from other geographical regions). Samples in bold indicate infection with trematode cercariae. *Biomphalaria pfeifferi* was used as an out-group. (B) ML phylogenetic tree of the current furcocercous cercaria and related trematodes based on a partial 28S rDNA sequence (838 bp). (C) ML phylogenetic tree of the current xiphidiocercaria and related trematodes based on a partial ITS2 sequence (349 bp). The bold taxa represent the cercaria samples obtained from the current study. Support values (ML bootstrap/NJ bootstrap/Bayesian posterior probabilities) are shown above the branches of the phylogenetic tree. At the branches of the tree, a dash (-) indicates less than 50% support value or that a certain grouping was not observed via that method of analysis. The red color on the map represents the locations found to be *I*. *exustus* infected with trematode cercariae. Abbreviations: NSN, Nakhon Sawan province; SKA, Songkhla province. The map was created using MapChart software’s free version licence https://www.mapchart.net/terms.html#licensing-maps, under a CC BY license with permission of Minas Giannekas (owner and creator of the map-making website mapchart.net).

Two out of 575 snails (0.35%) were found to be infected with cercariae (Table 1). Two types of cercarial stages of trematodes were identified as furcocercous cercaria (Fig 1B) and xiphidiocercaria (Fig 1C). Additionally, the furcocercous cercaria was released from one *I*. *exustus* that was collected from the irrigation canal in Nakhon Sawan province (Central Thailand). Moreover, the xiphidiocercaria was liberated from one *I*. *exustus* that was collected from the lotus pond in Songkhla province (Southern Thailand).

### Morphology of cercaria

A furcocercous cercaria was oval-elongated in shape. Its oral sucker was globular and situated at the terminal anterior part of the body, whereas the ventral sucker was located at three-fourths distance down the length of the body. In addition, it had one pair of eyespots. The tail was substantially longer than the body. Moreover, it had a dominant unique tail with two furcae, and each furca was obviously shorter than the tail stem (Fig 1B). The morphological characteristics of xiphidiocercaria were relatively small. Additionally, the body was oval-elongated in shape and unpigmented, and its oral sucker was globular and located in the subterminal region of the anterior part of the body, whereas the ventral sucker was situated in the middle of the body. This cercarial type had a dominant stylet present within the center of the oral sucker. The tail of this cercarial type was shorter than the body length (Fig 1C).

### Molecular characterization of cercaria

The partial sequence data from PCR fragments of the 28S rDNA and ITS2 region were used to identify phylogenetic relationships of furcocercous cercaria and xiphidiocercaria, respectively. Based on 838 bp of the 28S rDNA gene, 5 samples (GenBank accession nos. OP600054-OP600058) of furcocercous cercaria from one *I*. *exustus* showed a high degree of identity (99%) with *Euclinostomum* sp. (GenBank accession no. MW604803). The ML tree derived from all of the sequences of 28S rDNA from the present study was clustered with a clade of *Euclinostomum* sp. in the family Clinostomidae with branch support values of 90, 90, and 98% for the ML, NJ, and Bayesian posterior probabilities, respectively (Fig 1B). The ITS2 sequence (349 bp) from one sample of xiphidiocercaria (GenBank accession no. OP586621) from one *I*. *exustus* displayed 99% similarity to *Xiphidiocercariae* sp. (GenBank accession no. MW020045). A maximum likelihood tree showed that the sequence in the present study was clustered with a clade of *Xiphidiocercariae* sp. with high bootstrap support values for ML, NJ, and Bayesian posterior probabilities of 100% (Fig 1C).

### Molecular identification of *I*. *exustus*

To identify the *Indoplanorbis* species, 162 individual snails from 21 provinces in six regions of Thailand were randomly selected for genetic studies. Based on 569 bp of the COI gene, all 162 sequences (GenBank accession nos. OP588466-OP588627) in this study demonstrated the highest similarity (98–100%) with known COI sequences of *I*. *exustus* (GenBank accession nos. MH037077, MH037081, and MT274331). Moreover, the 16S rDNA gene (381 bp) from 162 specimens (GenBank accession nos. OP585918-OP586079) displayed 99–100% similarity with *I*. *exustus* (GenBank accession no. MH037103). In addition, the ITS1 sequences (GenBank accession nos. OP586437-OP586598) in this work showed 99–100% identity to *I*. *exustus* (GenBank accession no. MH037127) after a BLASTN search by using 600 bp of the ITS1 region.

### Genetic diversity of *I*. *exustus*

The cytochrome oxidase subunit I (COI) (569 bp), 16S ribosomal DNA (rDNA) (381 bp), internal transcribed spacer I (ITS1) (600 bp), and combined dataset of two mtDNA regions (950 bp) were genetically analyzed from the 162 individual *I*. *exustus* samples, which represented 21 populations from Thailand, along with sequences from other geographical regions in GenBank (Table 3).

Analysis of the mitochondrial COI was conducted on 206 samples. This analysis identified 48 haplotypes (C1-C48) with 170 variable nucleotide sites. Of these, 45 haplotypes were unique, and three were shared by multiple populations. Haplotype C25 was the most shared among the populations found in Benin, Gabon, Ivory Coast, and Malaysia. Additionally, the haplotype diversity in each population ranged from 0 to 1.0000, with an overall of 0.5803. The nucleotide diversity in each population ranged from 0 to 0.0816, with an overall of 0.0224 (Table 4). In addition, among the 162 samples in the present study, 23 haplotypes (C1-C23) were identified. Only the C1 and C4 haplotypes of *I*. *exustus* were infected with furcocercous cercaria and xiphidiocercariae, respectively. Haplotype C1 was widely distributed in 20 populations encompassing six geographic regions of Thailand, accounting for 79.0%. The haplotype diversity in each population in Thailand ranged from 0 to 0.7167, with an overall of 0.3756. Furthermore, the nucleotide diversity in each population ranged from 0 to 0.0046, with an overall of 0.0021 (Table 5).

**Table 4:** Diversity indices of COI and 16S rDNA sequences in the *I*. *exustus* populations from Thailand and other geographical regions.

Location	COI							16S rDNA						
	N	V	H	Sh	Uh	Hd ± SD	π ± SD	N	V	H	Sh	Uh	Hd ± SD	π ± SD
Thailand	162	60	23	2	21	0.3752 ± 0.0497	0.0021 ± 0.0014	162	3	4	1	3	0.1179 ± 0.0340	0.0003 ± 0.0005
Bangladesh	6	18	6	0	6	1.0000 ± 0.0962	0.0118 ± 0.0075	6	42	5	0	5	0.9333 ± 0.1217	0.0384 ± 0.0232
Benin	5	0	1	1	0	0.0000 ± 0.0000	0.0000 ± 0.0000	5	0	1	1	0	0.0000 ± 0.0000	0.0000 ± 0.0000
France	2	10	2	0	2	1.0000 ± 0.5000	0.0176 ± 0.0184	2	0	1	0	1	0.0000 ± 0.0000	0.0000 ± 0.0000
Gabon	1	0	1	1	0	NA	NA	1	0	1	1	0	NA	NA
India	1	0	1	0	1	NA	NA	1	0	1	0	1	NA	NA
Indonesia	1	0	1	0	1	NA	NA	1	0	1	1	0	NA	NA
Ivory Coast	1	0	1	1	0	NA	NA	1	0	1	1	0	NA	NA
Laos	1	0	1	1	0	NA	NA	1	0	1	1	0	NA	NA
Malaysia	4	5	2	2	0	0.6667 ± 0.2041	0.0058 ± 0.0045	4	0	1	1	0	0.0000 ± 0.0000	0.0000 ± 0.0000
Nepal	15	119	9	0	9	0.9048 ± 0.0544	0.0816 ± 0.0421	15	64	7	1	6	0.8476 ± 0.0648	0.0718 ± 0.0373
Oman	4	3	3	0	3	0.8333 ± 0.2224	0.0029 ± 0.0025	4	0	1	1	0	0.0000 ± 0.0000	0.0000 ± 0.0000
Philippines	1	0	1	1	0	NA	NA	1	0	1	1	0	NA	NA
Sri Lanka	1	0	1	0	1	NA	NA	1	0	1	0	1	NA	NA
Vietnam	1	0	1	0	1	NA	NA	1	0	1	1	0	NA	NA
Total	206	170	48	3	45	0.5803 ± 0.0431	0.0224 ± 0.0112	206	73	18	1	17	0.2954 ± 0.0429	0.0136 ± 0.0073

N = number of *I*. *exustus* examined, V = number of variable sites, H = number of haplotypes, Sh = Shared haplotypes, Uh = unique haplotypes, Hd = haplotype diversity, π = nucleotide diversity, NA = not calculated because of small sample size.

**Table 5:** Diversity indices of COI and 16S rDNA sequences in the *I*. *exustus* populations from 21 populations of Thailand.

Location	COI							16S rDNA						
	N	V	H	Sh	Uh	Hd ± SD	π ± SD	N	V	H	Sh	Uh	Hd ± SD	π ± SD
Uttaradit	1	0	1	1	0	NA	NA	1	0	1	1	0	NA	NA
Lamphun	6	5	2	2	0	0.3333 ± 0.2152	0.0029 ± 0.0023	6	0	1	1	0	0.0000 ± 0.0000	0.0000 ± 0.0000
Lampang	1	0	1	1	0	NA	NA	1	0	1	1	0	NA	NA
Chaiyaphum	8	9	2	1	1	0.2500 ± 0.1802	0.0039 ± 0.0027	8	0	1	1	0	0.0000 ± 0.0000	0.0000 ± 0.0000
Khon Kaen	2	0	1	1	0	0.0000 ± 0.0000	0.0000 ± 0.0000	2	0	1	1	0	0.0000 ± 0.0000	0.0000 ± 0.0000
Udon Thani	6	1	2	2	0	0.3333 ± 0.2152	0.0005 ± 0.0007	6	0	1	1	0	0.0000 ± 0.0000	0.0000 ± 0.0000
Phitsanulok	29	27	6	2	4	0.4729 ± 0.1098	0.0043 ± 0.0027	29	0	1	1	0	0.0000 ± 0.0000	0.0000 ± 0.0000
Sukhothai	3	0	1	1	0	0.0000 ± 0.0000	0.0000 ± 0.0000	3	0	1	1	0	0.0000 ± 0.0000	0.0000 ± 0.0000
Phichit	11	1	2	1	1	0.1818 ± 0.1436	0.0003 ± 0.0005	11	0	1	1	0	0.0000 ± 0.0000	0.0000 ± 0.0000
Phetchabun	1	0	1	1	0	NA	NA	1	0	1	1	0	NA	NA
Chai Nat	18	11	6	2	4	0.5621 ± 0.1342	0.0023 ± 0.0016	18	0	1	1	0	0.0000 ± 0.0000	0.0000 ± 0.0000
Sing Buri	16	11	6	2	4	0.7167 ± 0.0988	0.0046 ± 0.0029	16	1	2	1	1	0.1250 ± 0.1064	0.0003 ± 0.0005
Nakhon Sawan	9	1	2	1	1	0.2222 ± 0.1662	0.0003 ± 0.0005	9	0	1	1	0	0.0000 ± 0.0000	0.0000 ± 0.0000
Ang Thong	2	0	1	1	0	0.0000 ± 0.0000	0.0000 ± 0.0000	2	1	2	1	1	1.0000 ± 0.5000	0.0026 ± 0.0037
Ayuthaya	1	0	1	1	0	NA	NA	1	0	1	1	0	NA	NA
Nakhon Nayok	2	0	1	1	0	0.0000 ± 0.0000	0.0000 ± 0.0000	2	0	1	1	0	0.0000 ± 0.0000	0.0000 ± 0.0000
Tak	15	0	1	1	0	0.0000 ± 0.0000	0.0000 ± 0.0000	15	0	1	1	0	0.0000 ± 0.0000	0.0000 ± 0.0000
Chanthaburi	1	0	1	0	1	NA	NA	1	0	1	1	0	NA	NA
Chon Buri	10	4	4	2	2	0.5333 ± 0.1801	0.0014 ± 0.0012	10	0	1	1	0	0.0000 ± 0.0000	0.0000 ± 0.0000
Pattani	3	1	2	1	1	0.6667 ± 0.3143	0.0011 ± 0.0014	3	0	1	1	0	0.0000 ± 0.0000	0.0000 ± 0.0000
Songkhla	17	1	2	2	0	0.2206 ± 0.1208	0.0003 ± 0.0005	17	1	2	1	1	0.5294 ± 0.0450	0.0013 ± 0.0013
Total	162	60	23	4	19	0.3756 ± 0.0505	0.0021 ± 0.0015	162	3	4	1	3	0.1214 ± 0.0349	0.0003 ± 0.0005

N = number of *I*. *exustus* examined, V = number of variable sites, H = number of haplotypes, Sh = Shared haplotypes, Uh = unique haplotypes, Hd = haplotype diversity, π = nucleotide diversity, NA = not calculated because of small sample size.

Analysis of the 16S rDNA (206 sequences) identified 73 variable nucleotide sites, which were identified and classified into 18 haplotypes (S1-S18). Seventeen haplotypes were unique, and one was shared by multiple populations. Haplotype S2 was found in populations in Thailand, Benin, Gabon, Indonesia, Ivory Coast, Laos, Malaysia, Nepal, Oman, Philippines, and Vietnam. In addition, the haplotype diversity in each population ranged from 0 to 0.9333, with an overall of 0.2954. Nucleotide diversity in each population ranged from 0 to 0.0718, with an overall of 0.0136 (Table 4). Among the *I*. *exustus* (162 sequences) from Thailand, 4 haplotypes (S1-S4) were identified. Only the S2 haplotype was infected with furcocercous cercaria and xiphidiocercariae. Moreover, haplotype S2 was dispersed in all populations, accounting for 93.80% of the haplotypes. Haplotype diversity for Thailand ranged from 0 to 1.0000, with an overall of 0.1214, and nucleotide diversity ranged from 0 to 0.0026, with an overall of 0.0003 (Table 5).

Regarding the analysis of 950 bp of combined mtDNA sequences (COI + 16S rDNA) that were obtained from 206 individuals, a total of 53 haplotypes (CS1-CS53) were identified with 243 variable nucleotide sites. Of these, 50 haplotypes were unique, and three were shared by multiple populations. Haplotype CS28 was the most shared among the populations found in Benin, Gabon, Ivory Coast, and Malaysia. Moreover, haplotype diversity ranged from 0 to 1.0000, with an overall of 0.6419, and nucleotide diversity ranged from 0 to 0.0780, with an overall of 0.0189 (S1 Table). Among the samples from Thailand, 26 haplotypes (CS1-CS26) were identified. Only CS2 and CS4 haplotypes were infected with furcocercous cercaria and xiphidiocercariae, respectively. Additionally, the CS2 haplotype had the highest frequency and was dispersed in all populations (except for Chanthaburi Province), accounting for 72.80%. Haplotype diversity in Thailand ranged from 0 to 1.0000, with an overall of 0.4701, and nucleotide diversity ranged from 0 to 0.0029, with an overall of 0.0014 (S2 Table).

The sequence alignment of 194 *I*. *exustus* samples based on the ITS1 region showed 131 variable nucleotide sites. These nucleotide samples were classified into 22 haplotypes (T1-T22), of which there were twenty unique haplotypes and two shared haplotypes. Haplotype T1 was the most shared among the populations found in Thailand, Benin, Gabon, Ivory Coast, Malaysia, Oman, and Vietnam. Moreover, haplotype diversity ranged from 0 to 0.9000, with an overall of 0.3487, and nucleotide diversity ranged from 0 to 0.0815, with an overall of 0.0122 (S3 Table). Among the *I*. *exustus* in Thailand, 10 haplotypes (T1-T10) were identified. Only the T1 haplotype was infected with furcocercous cercaria and xiphidiocercariae. Haplotype T1 was dispersed in all populations (except Sukhothai Province), accounting for 89.50% of the haplotypes. Furthermore, haplotype diversity for Thailand ranged from 0 to 1.0000, with an overall of 0.2040, and nucleotide diversity ranged from 0 to 0.0033, with an overall of 0.0003 (S4 Table).

Comparatively, when examining the genetic variability among the genetic markers in *I*. *exustus* in Thailand, the genetic distances of COI, 16S rDNA, ITS1, and combined mtDNA sequences among *I*. *exustus* in Thailand were 0–5.82%, 0–0.53%, 0–0.67%, and 0–3.49%, respectively. When comparing the genetic distance between *I*. *exustus* infected with furcocercous cercaria from Nakhon Sawan province (I410NSN1) and *I*. *exustus* from other regions in Thailand, genetic differences based on four genetic markers (COI, 16S rDNA, ITS1, and combined mtDNA) were observed between both groups, with the highest genetic difference observed in the COI gene (0.09%). In contrast, between *I*. *exustus* infected with furcocercous cercaria from Nakhon Sawan province (I410NSN1) and *I*. *exustus* uninfected from Nakhon Sawan province, genetic differences were found in three genetic markers (COI, ITS1, and combined mtDNA), with the highest genetic difference observed in the ITS1 region (0.04%). Furthermore, the genetic difference between *I*. *exustus* infected with xiphidiocercaria from Songkhla province (I532SKA2) and *I*. *exustus* from other regions in Thailand was highest in the COI gene (0.09%), but no genetic difference of the four genetic markers between *I*. *exustus* infected with xiphidiocercaria from Songkhla province (I532SKA2) and *I*. *exustus* uninfected from Songkhla province was observed (Table 6). There was no genetic difference was observed between *I*. *exustus* infected with furcocercous cercaria (I410NSN1) and *I*. *exustus* infected with Xiphidiocercaria (I532SKA2).

**Table 6:** Pairwise genetic distances (%) among genetic markers of *I*. *exustus* samples.

Grouping for comparison	Genetic marker			
	COI	16S rDNA	Combined mtDNA	ITS1
Within *I*. *exustus* Thailand	0–5.82	0–0.53	0–3.49	0–0.67
*I*. *exustus* infected with Furcocercous cercaria Nakhon Sawan province (I410NSN1)- *I*. *exustus* Thailand	0.09	0.003	0.06	0.02
*I*. *exustus* infected with Furcocercous cercaria Nakhon Sawan province (I410NSN1)- *I*. *exustus* uninfected Nakhon Sawan province	0.02	0	0.01	0.04
*I*. *exustus* uninfected Nakhon Sawan province—*I*. *exustus* Thailand	0.12	0.0003	0.08	0.06
*I*. *exustus* infected with Xiphidiocercaria Songkhla province (I532SKA2)- *I*. *exustus* Thailand	0.09	0.003	0.06	0.02
*I*. *exustus* infected with Xiphidiocercaria Songkhla province (I532SKA2)- *I*. *exustus* uninfected Songkhla province	0	0	0	0
*I*. *exustus* uninfected Songkhla province- *I*. *exustus* Thailand	0.11	0.004	0.07	0.02
*I*. *exustus* infected with Furcocercous cercaria (I410NSN1)- *I*. *exustus* infected with Xiphidiocercaria (I532SKA2)	0	0	0	0

### Phylogenetic and network analyses of *I*. *exustus*

**Fig 2:**
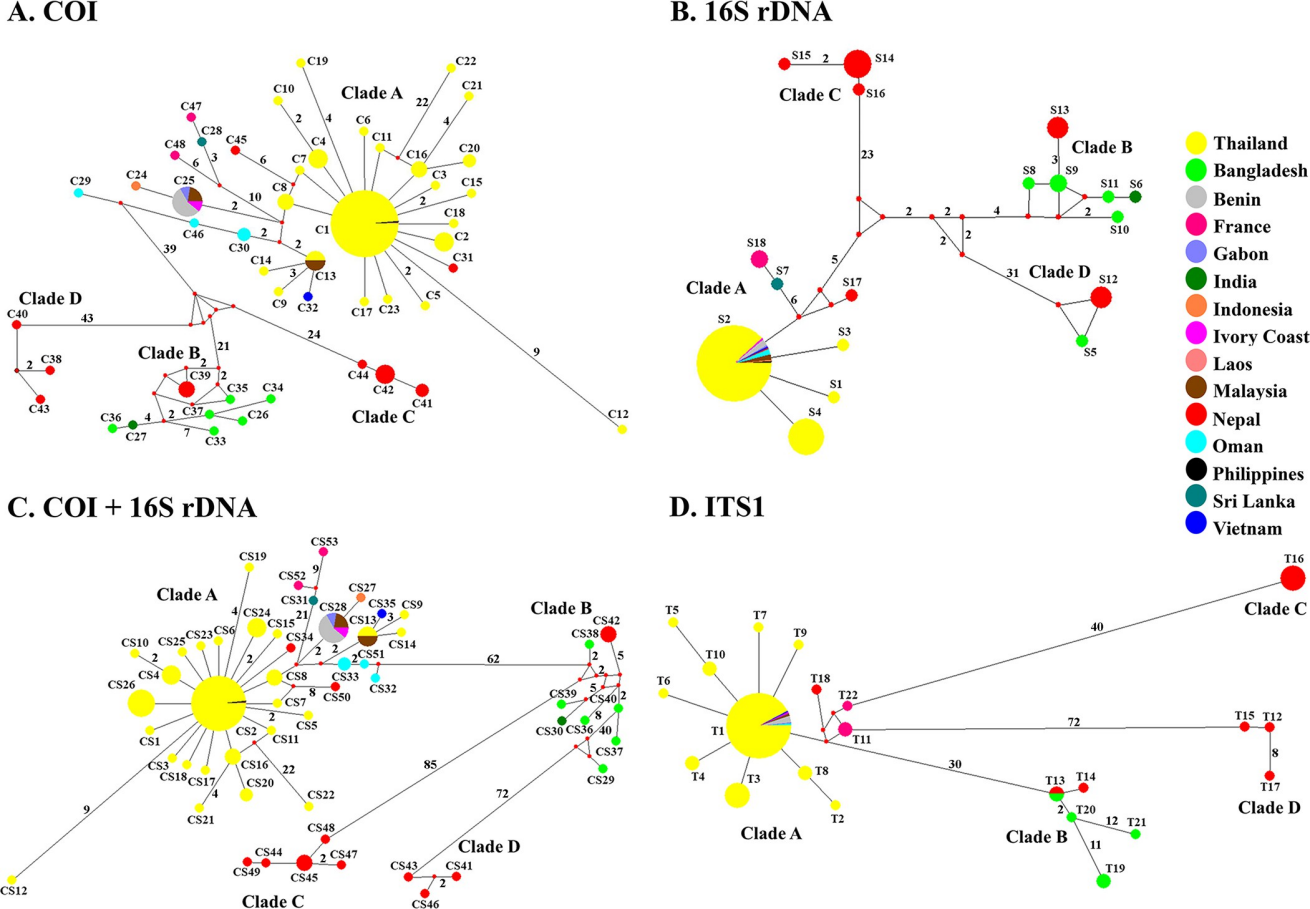
Median-joining haplotype networks of *I*. *exustus* from Thailand and other geographical regions. (A) Haplotype network of the COI gene. (B) Haplotype network of 16S rDNA. (C) Haplotype network of combined mtDNA. (D) Haplotype network of ITS1. Each haplotype is represented by a circle, and circle sizes are proportional to haplotype frequency. Colors indicate the geographic origin of the haplotypes. Number on each branch represents the mutational step number, while the branch with no number represents one mutation step. Median vectors (small red dots) represent ancestral haplotypes that are either not sampled or missing haplotypes.

The phylogenetic tree and haplotype network constructed from COI, 16S rDNA, ITS1, and combined mtDNA sequences demonstrated consistent results, which can be divided into four clades (A to D) (Figs 1A and 2 and S1–S3 Figs). *I*. *exustus* clade A was the largest group and the most widely distributed in many regions throughout the world. Furthermore, only clade A of *I*. *exustus* was infected with furcocercous cercaria and xiphidiocercariae (Fig 1A).

The MJ network mitochondrial COI sequences (569 bp) were obtained from 162 sequences from individual *I*. *exustus* of Thailand and 44 sequences from other geographical regions in GenBank. Clade A was the largest group (186 sequences, 34 haplotypes) and contained all of the haplotypes from Thailand (C1-C23) together with haplotypes from South Asia, Southeast Asia, Southwest Asia, West Africa, Central Africa, and the Caribbean region. Clades B, C, and D contained haplotypes from only South Asia (Figs 1A and 2A). The estimates of evolutionary divergence showed that the percentages “within clade” (between 0% and 7.05%) were lower than “among clade” (between 8.11% and 16.93%) (S5 Table).

The 16S rDNA sequences (381 bp) demonstrated topologies similar to those of the COI gene. Based on the 16S rDNA sequences, four genetically divergent clades were found among 162 individuals of *I*. *exustus* from Thailand and 44 sequences from other geographical regions. Levels of genetic differentiation among four clades were much higher than those within clades. The genetic divergence of the 16S rDNA within clades was 0–1.84%, and that among clades was 3.16–10.05% (S5 Table). Moreover, clade A included 7 haplotypes (186 sequences), with four haplotypes (S1-S4) from Thailand together with 3 other haplotypes from South Asia, Southeast Asia, Southwest Asia, West Africa, Central Africa, and the Caribbean region. Clades B, C, and D comprised haplotypes from only South Asia (Fig 2B and S1 Fig).

The MJ network of combined mtDNA sequences (950 bp) demonstrated four genetically distinct clades similar to those of COI and 16S rDNA sequences. Clade A consisted of 37 haplotypes (186 sequences) with haplotypes CS1-CS26 from Thailand and was closely related to haplotypes from South Asia, Southeast Asia, Southwest Asia, West Africa, Central Africa, and the Caribbean region. Clades B, C, and D contained haplotypes from only South Asia (Fig 2C and S2 Fig). The estimates of evolutionary divergence within clades and among clades are shown in S5 Table.

The MJ network based on ITS1 sequences (600 bp) identified four genetically distinct clades similar to those of COI and 16S rDNA and combined mtDNA (Fig 2D and S3 Fig). The estimates of evolutionary divergence within clades and among clades are shown in S5 Table.

**Fig 3:**
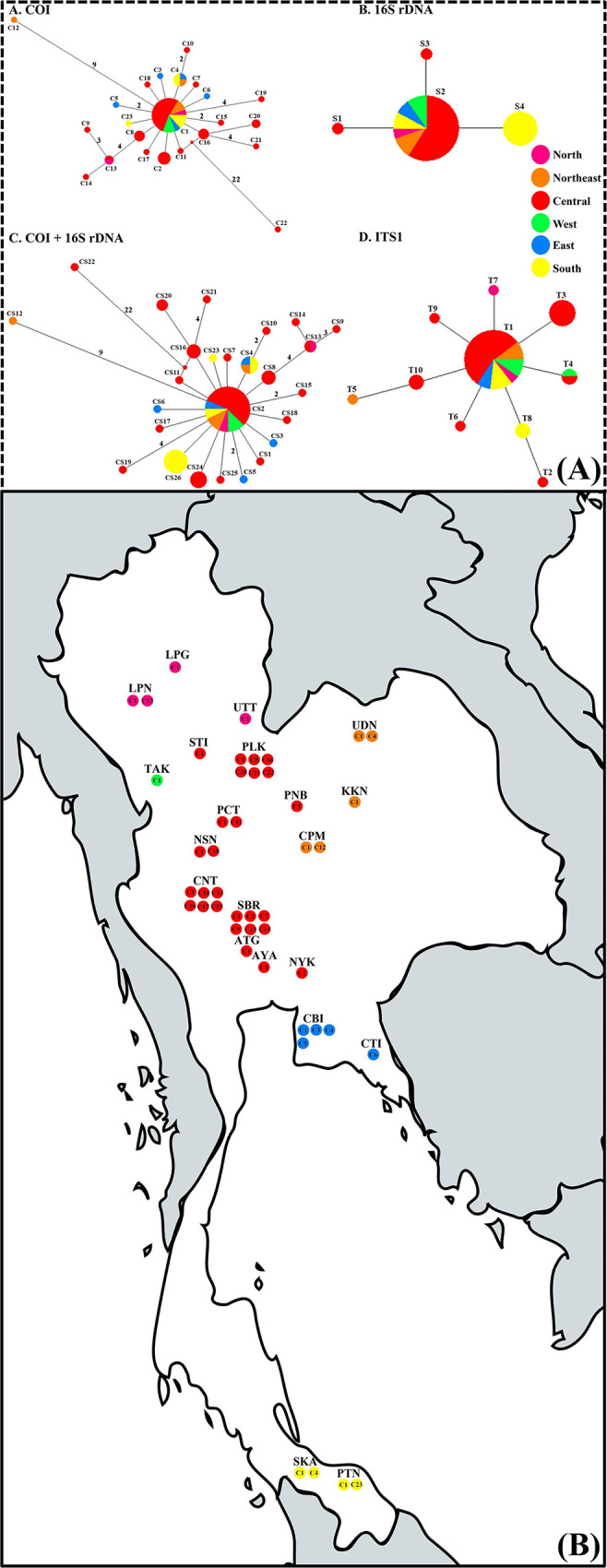
Distribution of the haplotype for *I*. *exustus* in Thailand. (A) Median joining network of 162 *I*. *exustus* in Thailand based on COI, 16S rDNA, combined mtDNA, and ITS1 sequences. Each haplotype is represented by a circle, and circle sizes are proportional to haplotype frequency. Colors indicate the geographic origin of the haplotypes. Number on each branch represents the mutational step number, while the branch with no number represents one mutation step. Median vectors (small red dots) represent ancestral haplotypes that are either not sampled or missing haplotypes. (B) Distribution of COI haplotypes of *I*. *exustus* in Thailand. Colors represent different regions: North = pink; Northeast = orange; Central = red; West = green; East = blue; and South = yellow. The map was created using MapChart software’s free version licence https://www.mapchart.net/terms.html#licensing-maps, under a CC BY license with permission of Minas Giannekas (owner and creator of the map-making website mapchart.net).

The haplotype network structure of *I*. *exustus* in Thailand represented a star-like phylogeny, with the most common haplotypes in the star’s center. For the MJ network in Thailand based on COI sequences, the most frequent haplotype C1 (128 sequences) was located at the center of the network and was widely distributed in 20 provinces in six regions. Moreover, based on the 16S rDNA gene, haplotype S2 (152 sequences) was the most widely distributed in 21 provinces in six regions. In addition, haplotype CS2 (118 sequences) of the MJ network based on the combined mtDNA was the most widely distributed in 20 provinces. Furthermore, based on ITS1 sequences, haplotype T1 (145 sequences) was the most widely distributed in 20 provinces in six regions of Thailand (Fig 3).

### Population genetic structure of *I*. *exustus*

**Fig 4:**
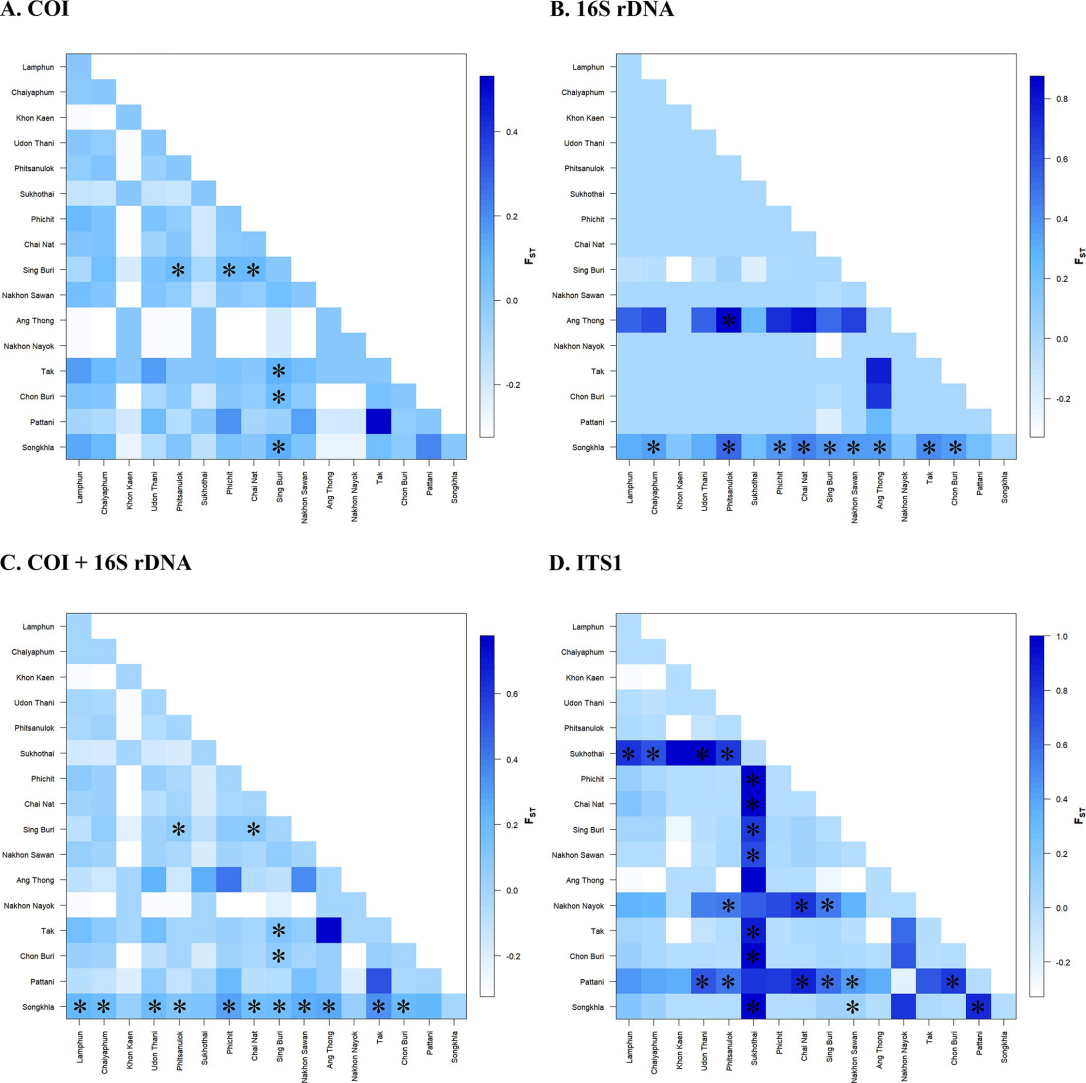
Graphical representation of the pairwise F_ST_ distance matrix among the *I*. *exustus* populations estimated from COI sequences (A), 16S rDNA sequences (B), combined mtDNA sequences (C), and ITS1 sequences (D). Statistically significant F_ST_ values are marked with an asterisk (*).

Genetic differentiation among populations (F_ST_) of *I*. *exustus* was analyzed based on COI, 16S rDNA, ITS1, and combined mtDNA. Population pairwise F_ST_ values for the COI, 16S rDNA, and combined mtDNA sequences of *I*. *exustus* from Thailand in 16 populations demonstrated that most populations were not genetically significantly different (Fig 4 and S6–S8 Tables). Moreover, the population pairwise F_ST_ values based on the ITS1 sequences demonstrated that most populations between Sukhothai and the other populations were significantly different (Fig 4 and S9 Table).

### Demographic history of *I*. *exustus*

**Fig 5:**
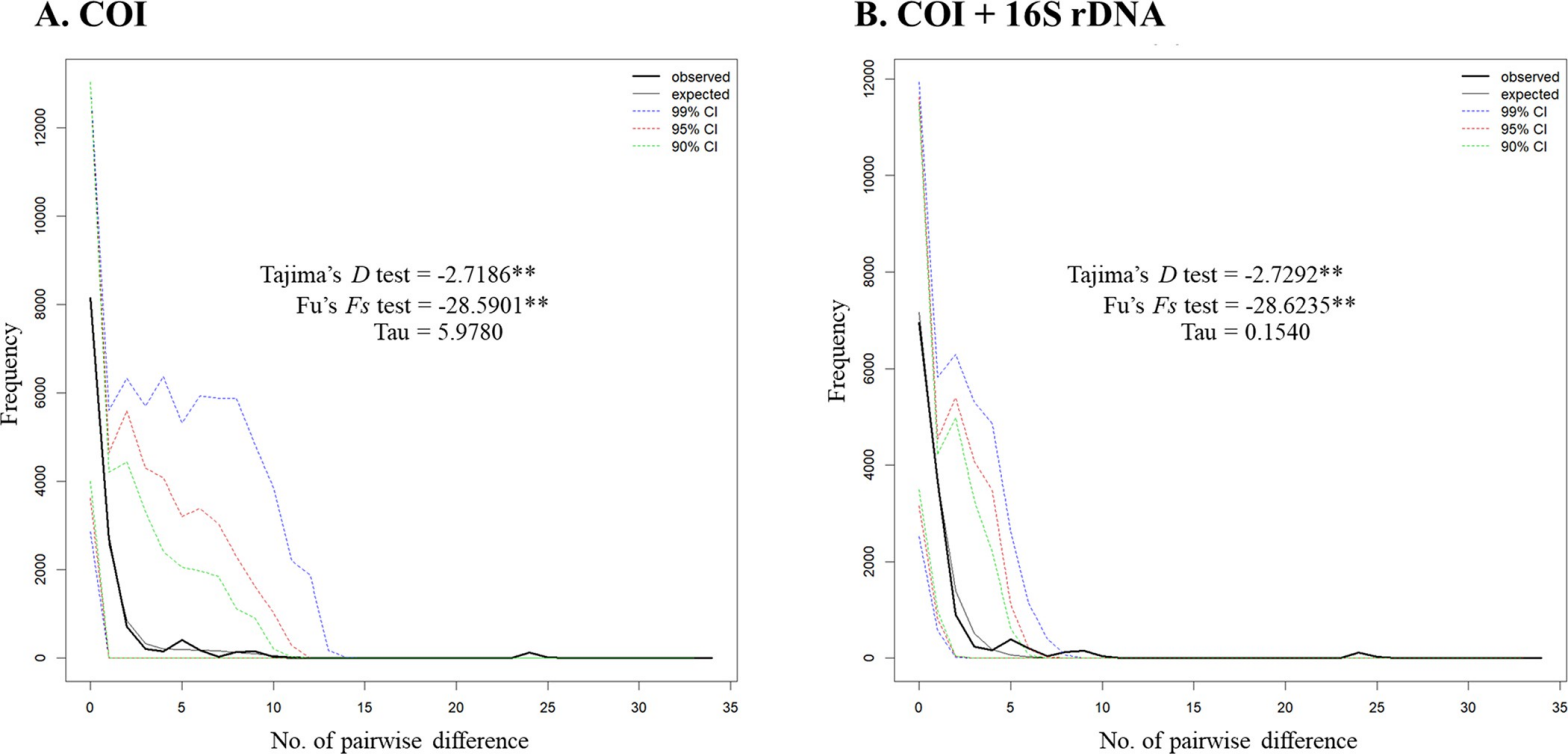
Mismatch distribution of the 162 sequences of *I*. *exustus* for Thailand demonstrating observed and expected pairwise differences based on the predictions of the sudden population expansion model. (A) Mismatch distribution of COI sequences of *I*. *exustus* consistent with the predictions of the sudden population expansion model (SSD = 0.0008, P = 0.6330; Harpending’s raggedness index = 0.2006, P = 0.7740). (B) Mismatch distribution of combined mtDNA sequences of *I*. *exustus* consistent with the predictions of the sudden population expansion model (SSD = 0.0034, P = 0.3980; Harpending’s raggedness index = 0.1121, P = 0.7920).

Mismatch distribution analysis demonstrated a unimodal mismatch graph (Fig 5), which was a characteristic of a recent population expansion. Both the sum-of-squares deviation and Harpending’s raggedness index were not significantly different from the simulated data under the sudden population expansion model (Fig 5). Population expansion was also supported by highly significant negative values of both Tajima’s *D* (−2.7186, P < 0.001 in COI; −2.7292, P < 0.001 in mtDNA) and Fu’s *F*_S_ (−28.5901, P < 0.001 in COI; −28.6235, P < 0.001 in mtDNA) tests.

## Discussion

The present study described the low infection rate of trematode cercariae in *I*. *exustus* across Thailand. A wide range of infective investigations of cercaria-stage trematodes in *I*. *exustus* have been performed in Thailand. Spotted studies have demonstrated high degrees of diversities of trematode larvae infected in *I*. *exustus* [6, 42]. Our current study observed an overall 0.35% prevalence of cercarial infection in *I*. *exustus* collected from several habitats from 21 provinces in six regions of Thailand. The overall prevalence of infection was lower than in previous reports. Chontananarth et al. (2017) [43] surveyed the cercarial stages of trematodes in *I*. *exustus* from Nakhon Nayok province and reported a prevalence of 0.64%, whereas Wiroonpan et al. (2021) [42] demonstrated a 7.39% overall prevalence of trematode cercariae infection in *I*. *exustus* from Bangkok province. Most recently, Krailas et al. (2022) [6] reported of a 2.05% prevalence of cercariae-infected *I*. *exustus*, which was collected from Songkhla province of Thailand. The low cercarial infection rate may reflect the low diversity of secondary intermediate hosts and definitive hosts because of the lack of suitable habitats in urbanized areas. According to McKinney (2008) [44], localities with extremely urbanized areas tend to have low species diversity of mammals, reptiles, amphibians, and invertebrates. In addition, lower levels of certain anthropogenic activities, such as open-field defecation and urination, as well as a comparatively low proportion of livestock farming, can lead to low trematode infection rates in freshwater snails [45]. Another aspect (the low infection rates) could also be due to low parasite pressure due to infrequent miracidia and snail contact or to the acquisition of a level of resistance to infection by hosts [46]. Based on field observations, the *I*. *exustus* snail was commonly found to exist near or at the water surface level by attaching to floating aquatic plants or floating objects that move with the water current. Thus, it is possible that the low cercarial infection rate that was observed in this study results from a low probability of encounter between this snail species and miracidium-stage trematodes.

In the current study, only two cercarial types (furcocercous cercaria and xiphidiocercariae) were detected in *I*. *exustus* collected from Nakhon Sawan and Songkhla provinces, respectively. The external features of the furcocercous cercaria in the present investigation were consistent with the characteristics of furcocercous cercaria emerging from *I*. *exustus* [6]. The morphological features of the xiphidiocercariae in the current study were also consistent with the characteristics of the xiphidiocercaria collected from *Bithynia siamensis siamensis* [42]. In this study, the furcocercous cercaria were molecularly classified and closely clustered with a clade of *Euclinostomum* sp. in the family Clinostomidae. The family Clinostomidae is one of the parasites belonging to the digenetic trematode, with a life cycle requiring two intermediate hosts (snail and fish or frog) and one definitive host (bird). Adult flukes live in the oral cavities, pharynxes, and esophagi of reptiles and fish-eating birds (especially piscivorous birds) [47, 48]. At present, *Euclinostomum* sp. has never been reported to be involved in human diseases. For xiphidiocercariae, this cercaria was molecularly classified as having a close relationship to *Xiphidiocercariae* sp. with high bootstrap support values for ML, NJ, and Bayesian posterior probabilities of 100%. This type of cercaria has been previously observed in only Prosobranch snail (*B*. *s*. *siamensis*) collected from Bangkok province of Thailand [42]. However, there have been no reports on the pathogenesis and lifecycle of this cercaria.

In the present study, genetic characterization based on sequencing of the COI, 16S rDNA, ITS1, and combined mtDNA genes of *I*. *exustus* collected from across Thailand was performed with sequences from other geographical regions in GenBank. These genetic markers provided congruent results from the phylogenetic tree and haplotype network, which divided *I*. *exustus* into four different clades (A to D). For the phylogenetic tree and haplotype network based on COI sequences, clade A was the largest group containing haplotypes from all samples of Thailand together with South Asia, Southeast Asia, Southwest Asia, West Africa, Central Africa, and Caribbean region. Clade B contained all of the haplotype samples from South Asia, whereas clades C and D consisted of only samples from Nepal in South Asia. These results indicated that *I*. *exustus* clade A is the most widely distributed clade in many regions throughout the world. Genetic groups that were classified in our study were in concordance with all previous studies [3, 5, 15]. Mouahid et al. (2018) [3] revealed a phylogenetic tree of *I*. *exustus* divided into four clades (A-D). Clade A was specifically found in South Asia, clades B and C occurred in Southeast Asia and South Asia, and clade D occurred throughout a wide geographical range encompassing South Asia, Southeast Asia, Southwest Asia, West Africa, Central Africa, and the Caribbean region. The very high level of genetic divergence between the four clades ranged from 8.11% to 16.93% for COI, 3.16% to 10.05% for 16S rDNA, 6.34% to 13.74% for combined mtDNA, and 2.38% to 9.01% for ITS1. *Indoplanorbis exustus* was likely a complex of cryptic species, which was proposed by Devkota et al. (2015) [5], Gauffre‐Autelin et al. (2017) [15], and Saijuntha et al. (2021) [4].

With regard to the genetic variation of *I*. *exustus*, we examined the genetic variation of *I*. *exustus* covering 31 localities in 21 provinces in six regions of Thailand. *I*. *exustus* in Thailand showed high genetic divergence with 23 haplotypes (COI sequence), which was congruent with a previous report by Saijuntha et al. (2021) [4]. These scholars reported of a high genetic divergence with 21 haplotypes of *I*. *exustus* in Thailand based on the COI sequence. In addition, our results demonstrated that all of the haplotypes of *I*. *exustus* in Thailand (based on COI, 16S rDNA, ITS1, and combined mtDNA) belonged to clade A, which was the most common genetic group distributed worldwide. However, there were several unique haplotypes found in specific localities, thus leading to genetic differences between many localities. The high rate of self-fertilization in *I*. *exustus* [16] may lead to levels of population genetic differences because self-fertility allows for a single individual to rapidly establish populations in new habitats [49]. In addition, the different rivers in several areas of Thailand may also affect genetic differences and the genetic structure of *I*. *exustus* because the water flow was limited or not possible between various catchments. This would subsequently lead to limited gene flow and migration between the *I*. *exustus* populations across catchments, thus resulting in high degrees of genetic differences between areas. This phenomenon has been previously observed in other freshwater snails in Thailand, such as *Bithynia siamensis* and *Hydrobioides nassa* [50, 51].

Population genetic structure analysis of *I*. *exustus* in Thailand demonstrated that most comparisons were not genetically significantly different. This suggests that the gene flow within the *I*. *exustus* population in Thailand may elicit genetic homogeneity [52]. In addition, the MJ network analysis demonstrated that most specimens shared the central haplotype, with almost all of the remaining haplotypes connected to this central haplotype with a short branch length. The overall pattern resembles a star-like shape, which is characteristic of the recent demographic expansion of populations [53]. This effect was also supported by the unimodal mode of the mismatch distribution graph and highly negative values of both Tajima’s *D* and Fu’s *Fs* tests. Therefore, it is probable that the *I*. *exustus* snail is another freshwater snail invasive species in Thailand.

Our study was in concordance with the previous studies of Saijuntha et al. (2021) [4], which revealed that *I*. *exustus* in Thailand was commonly found in paddy fields, canals, and swamps, which were widely distributed in more than 30 provinces covering six regions of Thailand. This study also demonstrated that *I*. *exustus* in Thailand shared haplotypes throughout multiple populations, despite some areas being geographically distant localities. The presence of *I*. *exustus* far from its “original” geographic region is possibly linked to human activities of trading aquatic plants on which *I*. *exustus* can be attached. This scenario is one of the main sources of migration and gene flow of *I*. *exustus* in Thailand and worldwide [54].

*I*. *exustus* is a hermaphroditic invasive snail species with high fecundity. Within one year of introduction, the snail can colonize habitats with well-established populations of other pulmonate snails [54]. The capacity for self-fertilization and high fecundity likely underlies the invasive potential of the species [55] and may be important for the distribution of the parasite, as it is the natural intermediate host of many trematodes, such as *Schistosoma indicum*, *S*. *nasale*, *S*. *spindale*, and *Echinostoma* spp. [5, 6]. Therefore, when the vector of parasitosis is present in a geographical area, nothing excludes the emergence of the disease if the parasite is introduced [56].

Remarkably, our study found that only *I*. *exustus* clade A was infected with trematode cercariae, which is the most widely distributed clade in many regions throughout the world. In contrast to a previous study, Devkota et al. (2015) [5] reported of the existence of a snail-host complex and classified *I*. *exustus* into four different lineages. Lineage I included *I*. *exustus* infected with echinostome and *Schistosoma* sp. Lineage II comprised snails infected with xiphidiocercariae, strigeids, and sanguinicolid cercariae. Lineage III consisted of snails positive for *S*. *nasale*, and lineage IV comprised snails infected with strigeids, sanguinicolids, and xiphidiocercariae, *S*. *indicum*, *S*. *spindale*, *S*. *nasale*, and *Schistosoma* sp. The genetic composition of intermediate snail hosts could be an important factor determining the establishment success of parasites in a certain area [57]. At the molecular level, this scenario complies with the hypothesis of a matching phenotype model, wherein the interactions between parasite antigens and host immune receptors during the early stages of the infection determine the success or failure of the infection [58]. Snails compatible with parasites have little or no immunopathic responses upon infection, thus allowing parasites to successfully establish and develop [59]. However, the simultaneous characterization of host and parasite population genetic structure from natural populations is essential to understand coevolutionary trajectories [60].

## Conclusion

In conclusion, we provide comprehensive data on cercarial infection and *I*. *exustus* snail distribution in Thailand. The snails from two provinces were infected with cercarial trematodes, and two types of cercarial stages were identified, including furcocercous cercaria and xiphidiocercaria. The haplotype network structure of *I*. *exustus* of Thailand has a star-like phylogeny, which is a characteristic of the recent demographic expansion of populations. This scenario is also supported by the unimodal mode of the mismatch distribution graph and highly negative values of both Tajima’s D and Fu’s Fs tests. Therefore, it is probable that the *I*. *exustus* snail is another freshwater snail invasive species in Thailand. In the future, more samples and a broader distribution of sampling sites are essential initial steps to investigate the genetic distribution patterns of *I*. *exustus*. Additionally, evaluating intricate causal connections between trematode infections and genetic alterations in snail hosts is a critical aspect that should not be disregarded.

## Supporting information

S1 Fig Maximum likelihood tree of 16S rDNA sequences (381 bp) of 18 haplotypes generated from 206 sequences of *I*. *exustus* (162 sequences from Thailand and 44 sequences from other geographical regions). Support values (ML bootstrap/NJ bootstrap/Bayesian posterior probabilities) are shown above the branches. At the branches of the tree, a dash (-) indicates less than 50% support value or that a certain grouping was not seen by that method of analysis. Samples in bold indicate infected with trematode cercariae. *Biomphalaria pfeifferi* was used as an out-group. Abbreviations: NSN, Nakhon Sawan Province; SKA, Songkhla Province.
(TIF)

S2 Fig Maximum likelihood tree of the combined mitochondrial genes of COI and 16S rDNA (950 bp) of 53 haplotypes generated from 206 sequences of *I*. *exustus* (162 sequences from Thailand and 44 sequences from other geographical regions). Support values (ML bootstrap/NJ bootstrap/Bayesian posterior probabilities) are shown above the branches. At the branches of the tree, a dash (-) indicates less than 50% support value or that a certain grouping was not seen by that method of analysis. Samples in bold indicate infected with trematode cercariae. *Biomphalaria pfeifferi* was used as an out-group. Abbreviations: NSN, Nakhon Sawan Province; SKA, Songkhla Province.
(TIF)

S3 Fig Maximum likelihood tree of ITS1 sequences (600 bp) of 22 haplotypes generated from 194 sequences of *I*. *exustus* (162 sequences from Thailand and 32 sequences from other geographical regions). Support values (ML bootstrap/NJ bootstrap/Bayesian posterior probabilities) are shown above the branches. At the branches of the tree, a dash (-) indicates less than 50% support value or that a certain grouping was not seen by that method of analysis. Samples in bold indicate infected with trematode cercariae. *Biomphalaria pfeifferi* was used as an out-group. Abbreviations: NSN, Nakhon Sawan Province; SKA, Songkhla Province.
(TIF)

S1 Table Diversity indices of the combined mtDNA in the *I*. *exustus* populations from Thailand and other geographical regions. (PDF)

S2 Table Diversity indices of the combined mt DNA in the *I*. *exustus* populations from 21 provinces of Thailand. (PDF)

S3 Table Diversity indices of ITS1 sequences in the *I*. *exustus* populations from Thailand and other geographical regions. (PDF)

S4 Table Diversity indices of ITS1 sequences in the *I*. *exustus* populations from 21 provinces of Thailand. (PDF)

S5 Table Estimates of genetic differences (%) within (bold) and among clades of *I*. *exustus* based on COI, 16S rDNA, combined mtDNA, and ITS1 sequences. (PDF)

S6 Table Population pairwise F_ST_ between 16 populations of *I*. *exustus* based on mitochondrial cytochrome c oxidase I gene sequences. (PDF)

S7 Table Population pairwise F_ST_ between 16 populations of *I*. *exustus* based on mitochondrial 16S rDNA sequences. (PDF)

S8 Table Population pairwise F_ST_ between 16 populations of *I*. *exustus* of the combined mtDNA sequences. (PDF)

S9 Table Population pairwise F_ST_ between 16 populations of *I*. *exustus* based on ITS1 sequences. (PDF)
