# Supplementary figures and images for: Population genetic structure of Indoplanorbis exustus (Gastropoda: Planorbidae) in Thailand and its infection with trematode cercariae
Source: PLoS One. 2024 Jan 26;19(1):e0297761. doi: 10.1371/journal.pone.0297761 (PMC10817173; doi:10.1371/journal.pone.0297761)

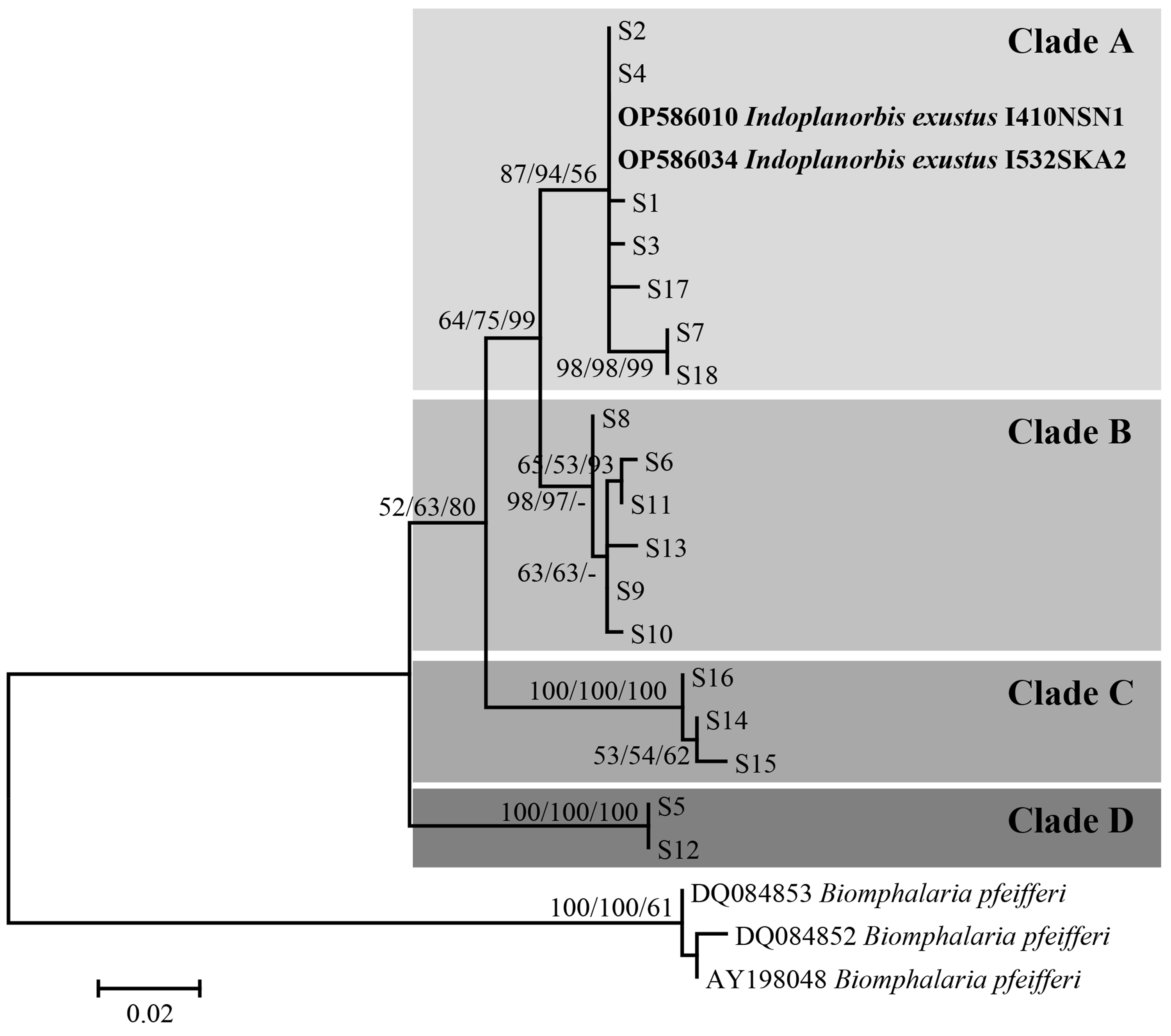

Supplement: S1 Fig — Support values (ML bootstrap/NJ bootstrap/Bayesian posterior probabilities) are shown above the branches. At the branches of the tree, a dash (-) indicates less than 50% support value or that a certain grouping was not seen by that method of analysis. Samples in bold indicate infected with trematode cercariae. Biomphalaria pfeifferi was used as an out-group. Abbreviations: NSN, Nakhon Sawan Province; SKA, Songkhla Province. (TIF) [file pone.0297761.s001.tif]

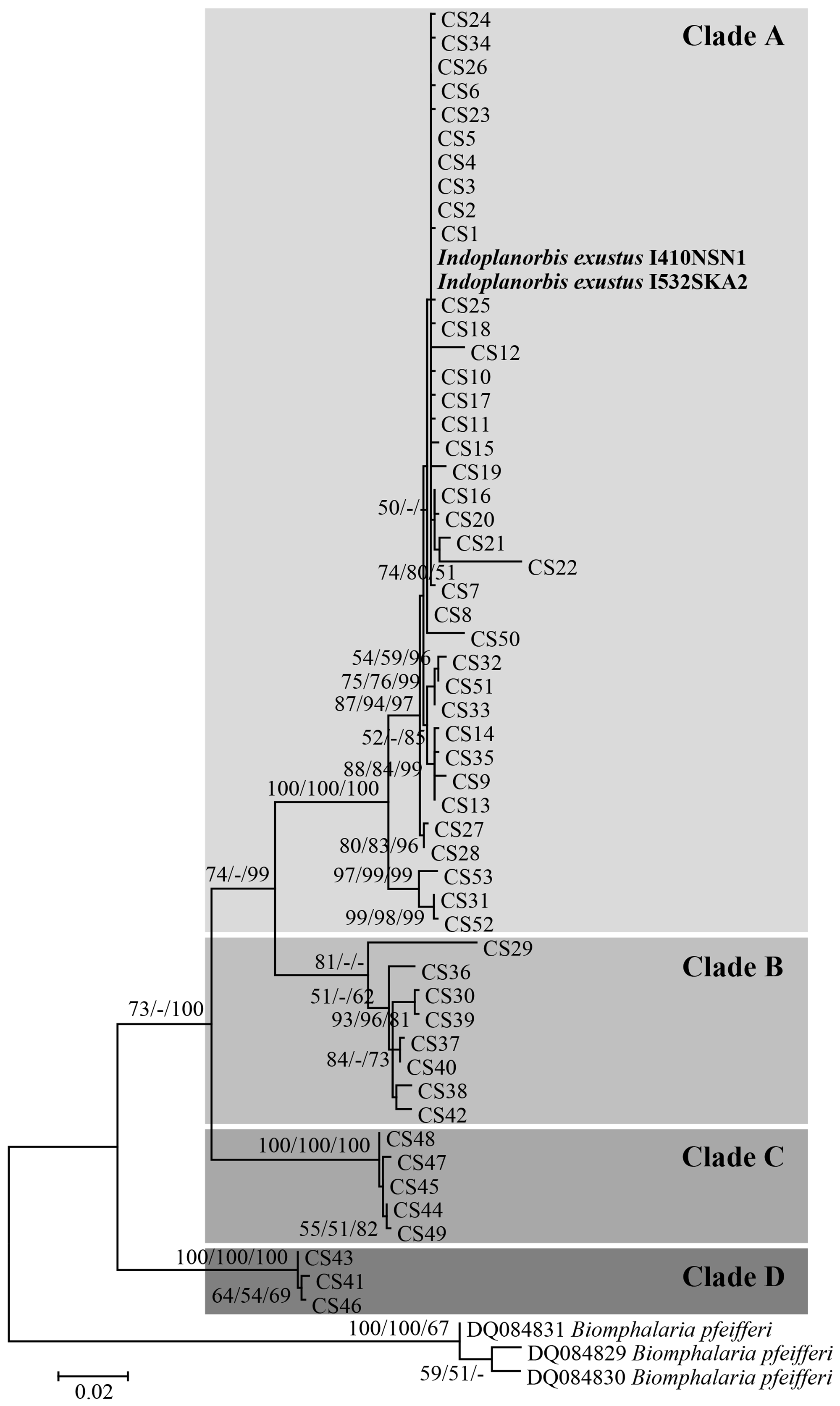

Supplement: S2 Fig — Support values (ML bootstrap/NJ bootstrap/Bayesian posterior probabilities) are shown above the branches. At the branches of the tree, a dash (-) indicates less than 50% support value or that a certain grouping was not seen by that method of analysis. Samples in bold indicate infected with trematode cercariae. Biomphalaria pfeifferi was used as an out-group. Abbreviations: NSN, Nakhon Sawan Province; SKA, Songkhla Province. (TIF) [file pone.0297761.s002.tif]

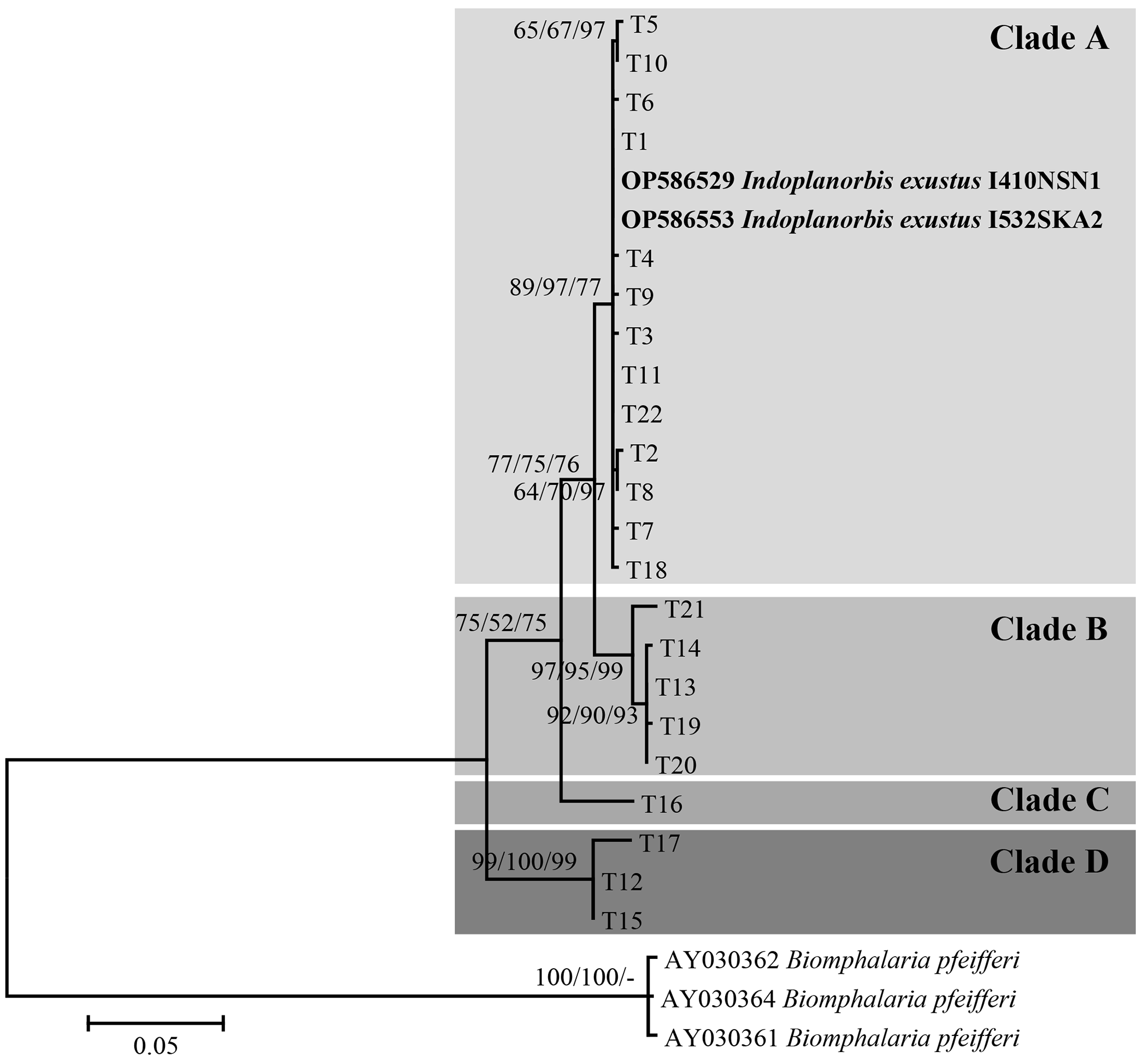

Supplement: S3 Fig — Support values (ML bootstrap/NJ bootstrap/Bayesian posterior probabilities) are shown above the branches. At the branches of the tree, a dash (-) indicates less than 50% support value or that a certain grouping was not seen by that method of analysis. Samples in bold indicate infected with trematode cercariae. Biomphalaria pfeifferi was used as an out-group. Abbreviations: NSN, Nakhon Sawan Province; SKA, Songkhla Province. (TIF) [file pone.0297761.s003.tif]
