# Supplementary material for: Population genetic structure of Indoplanorbis exustus (Gastropoda: Planorbidae) in Thailand and its infection with trematode cercariae
Source: PLoS One. 2024 Jan 26;19(1):e0297761. doi: 10.1371/journal.pone.0297761 (PMC10817173; doi:10.1371/journal.pone.0297761)
Supplement: S1 Table — (PDF) [file pone.0297761.s004.pdf]

S1 Table. Diversity indices of the combined mtDNA in the *I. exustus* populations from Thailand and other geographical regions.

Location	Number of *I. exustus* examined	Number of variable sites	Number of haplotypes	Shared haplotypes	Unique haplotypes	Haplotype diversity (h), mean ± SD	Nucleotide diversity (π), mean ± SD
Thailand	162	63	26	2	24	0.4670 ± 0.0496	0.0013 ± 0.0009
Bangladesh	6	61	6	0	6	1.0000 ± 0.0962	0.0231 ± 0.0137
Benin	5	0	1	1	0	0.0000 ± 0.0000	0.0000 ± 0.0000
France	2	10	2	0	2	1.0000 ± 0.5000	0.0105 ± 0.0111
Gabon	1	0	1	1	0	NA	NA
India	1	0	1	0	1	NA	NA
Indonesia	1	0	1	0	1	NA	NA
Ivory Coast	1	0	1	1	0	NA	NA
Laos	1	0	1	1	0	NA	NA
Malaysia	4	5	2	2	0	0.6667 ± 0.2041	0.0035 ± 0.0027
Nepal	15	183	11	0	11	0.9429 ± 0.0454	0.0780 ± 0.0399
Oman	4	3	3	0	3	0.8333 ± 0.2224	0.0017 ± 0.0015
Philippines	1	0	1	1	0	NA	NA
Sri Lanka	1	0	1	0	1	NA	NA
Vietnam	1	0	1	0	1	NA	NA
Total	206	243	53	3	50	0.6419 ± 0.0406	0.0189 ± 0.0093

NA = not calculated because of small sample size.
