# Supplementary material for: Population genetic structure of Indoplanorbis exustus (Gastropoda: Planorbidae) in Thailand and its infection with trematode cercariae
Source: PLoS One. 2024 Jan 26;19(1):e0297761. doi: 10.1371/journal.pone.0297761 (PMC10817173; doi:10.1371/journal.pone.0297761)
Supplement: S2 Table — (PDF) [file pone.0297761.s005.pdf]

S2 Table. Diversity indices of the combined mt DNA in the *I. exustus* populations from 21 provinces of Thailand.

Location	Number of *I. exustus* examined	Number of variable sites	Number of haplotypes	Shared haplotypes	Unique haplotypes	Haplotype diversity (h), mean ± SD	Nucleotide diversity (π), mean ± SD
Uttaradit	1	0	1	1	0	NA	NA
Lamphun	6	5	2	2	0	0.3333 ± 0.2152	0.0017 ± 0.0013
Lampang	1	0	1	1	0	NA	NA
Chaiyaphum	8	9	2	1	1	0.2500 ± 0.1802	0.0023 ± 0.0016
Khon Kaen	2	0	1	1	0	0.0000 ± 0.0000	0.0000 ± 0.0000
Udon Thani	6	1	2	2	0	0.3333 ± 0.2152	0.0003 ± 0.0004
Phitsanulok	29	27	6	2	4	0.4729 ± 0.1098	0.0026 ± 0.0016
Sukhothai	3	0	1	1	0	0.0000 ± 0.0000	0.0000 ± 0.0000
Phichit	11	1	2	1	1	0.1818 ± 0.1436	0.0001 ± 0.0003
Phetchabun	1	0	1	1	0	NA	NA
Chai Nat	18	11	6	2	4	0.5621 ± 0.1342	0.0013 ± 0.0010
Sing Buri	16	12	7	2	5	0.7750 ± 0.0876	0.0029 ± 0.0018
Nakhon Sawan	9	1	2	1	1	0.2222 ± 0.1662	0.0002 ± 0.0003
Ang Thong	2	1	2	1	1	1.0000 ± 0.5000	0.0011 ± 0.0014
Ayuthaya	1	0	1	1	0	NA	NA
Nakhon Nayok	2	0	1	1	0	0.0000 ± 0.0000	0.0000 ± 0.0000
Tak	15	0	1	1	0	0.0000 ± 0.0000	0.0000 ± 0.0000
Chanthaburi	1	0	1	0	1	NA	NA
Chon Buri	10	4	4	2	2	0.5333 ± 0.1801	0.0008 ± 0.0007
Pattani	3	1	2	1	1	0.6667 ± 0.3143	0.0007 ± 0.0008
Songkhla	17	2	3	2	1	0.6324 ± 0.0661	0.0008 ± 0.0006
Total	162	63	26	4	22	0.4701 ± 0.0503	0.0014 ± 0.0009

NA = not calculated because of small sample size.
