# Supplementary material for: Population genetic structure of Indoplanorbis exustus (Gastropoda: Planorbidae) in Thailand and its infection with trematode cercariae
Source: PLoS One. 2024 Jan 26;19(1):e0297761. doi: 10.1371/journal.pone.0297761 (PMC10817173; doi:10.1371/journal.pone.0297761)
Supplement: S3 Table — (PDF) [file pone.0297761.s006.pdf]

S3 Table. Diversity indices of ITS1 sequences in the *I. exustus* populations from Thailand and other geographical regions.

Location	Number of *I. exustus* examined	Number of variable sites	Number of haplotypes	Shared haplotypes	Unique haplotypes	Haplotype diversity (h), mean ± SD	Nucleotide diversity (π), mean ± SD
Thailand	162	9	10	1	9	0.1981 ± 0.0422	0.0003 ± 0.0005
Bangladesh	5	25	4	1	3	0.9000 ± 0.1610	0.0201 ± 0.0128
Benin	5	0	1	1	0	0.0000 ± 0.0000	0.0000 ± 0.0000
France	3	2	2	0	2	0.6667 ± 0.3143	0.0022 ± 0.0022
Gabon	1	0	1	1	0	NA	NA
Ivory Coast	1	0	1	1	0	NA	NA
Malaysia	2	0	1	1	0	0.0000 ± 0.0000	0.0000 ± 0.0000
Nepal	12	116	7	1	6	0.7727 ± 0.1276	0.0815 ± 0.0427
Oman	2	0	1	1	0	0.0000 ± 0.0000	0.0000 ± 0.0000
Vietnam	1	0	1	1	0	NA	NA
Total	194	131	22	2	20	0.3487 ± 0.0453	0.0122 ± 0.0063

NA = not calculated because of small sample size.
