# Supplementary material for: Population genetic structure of Indoplanorbis exustus (Gastropoda: Planorbidae) in Thailand and its infection with trematode cercariae
Source: PLoS One. 2024 Jan 26;19(1):e0297761. doi: 10.1371/journal.pone.0297761 (PMC10817173; doi:10.1371/journal.pone.0297761)
Supplement: S4 Table — (PDF) [file pone.0297761.s007.pdf]

S4 Table. Diversity indices of ITS1 sequences in the *I. exustus* populations from 21 provinces of Thailand.

Location	Number of *I. exustus* examined	Number of variable sites	Number of haplotypes	Shared haplotypes	Unique haplotypes	Haplotype diversity (h), mean ± SD	Nucleotide diversity (π), mean ± SD
Uttaradit	1	0	1	1	0	NA	NA
Lamphun	6	1	2	1	1	0.3333 ± 0.2152	0.0005 ± 0.0007
Lampang	1	0	1	1	0	NA	NA
Chaiyaphum	8	2	2	1	1	0.2500 ± 0.1802	0.0008 ± 0.0008
Khon Kaen	2	0	1	1	0	0.0000 ± 0.0000	0.0000 ± 0.0000
Udon Thani	6	0	1	1	0	0.0000 ± 0.0000	0.0000 ± 0.0000
Phitsanulok	29	3	4	2	2	0.2586 ± 0.1042	0.0004 ± 0.0005
Sukhothai	3	0	1	1	0	0.0000 ± 0.0000	0.0000 ± 0.0000
Phichit	11	0	1	1	0	0.0000 ± 0.0000	0.0000 ± 0.0000
Phetchabun	1	0	1	1	0	NA	NA
Chai Nat	18	0	1	1	0	0.0000 ± 0.0000	0.0000 ± 0.0000
Sing Buri	16	1	2	2	0	0.2333 ± 0.1256	0.0003 ± 0.0005
Nakhon Sawan	9	2	3	2	1	0.4167 ± 0.1907	0.0007 ± 0.0008
Ang Thong	2	0	1	1	0	0.0000 ± 0.0000	0.0000 ± 0.0000
Ayuthaya	1	0	1	1	0	NA	NA
Nakhon Nayok	2	2	2	1	1	1.0000 ± 0.5000	0.0033 ± 0.0041
Tak	15	1	2	2	0	0.1333 ± 0.1123	0.0002 ± 0.0003
Chanthaburi	1	0	1	1	0	NA	NA
Chon Buri	10	0	1	1	0	0.0000 ± 0.0000	0.0000 ± 0.0000
Pattani	3	1	2	1	1	0.6667 ± 0.3143	0.0011 ± 0.0013
Songkhla	17	0	1	1	0	0.0000 ± 0.0000	0.0000 ± 0.0000
Total	162	9	10	3	7	0.2040 ± 0.0433	0.0003 ± 0.0005

NA = not calculated because of small sample size.
