# Supplementary material for: Population genetic structure of Indoplanorbis exustus (Gastropoda: Planorbidae) in Thailand and its infection with trematode cercariae
Source: PLoS One. 2024 Jan 26;19(1):e0297761. doi: 10.1371/journal.pone.0297761 (PMC10817173; doi:10.1371/journal.pone.0297761)
Supplement: S5 Table — (PDF) [file pone.0297761.s008.pdf]

S5 Table. Estimates of genetic differences (%) within (bold) and among clades of *I. exustus* based on COI, 16S rDNA, combined mtDNA, and ITS1 sequences.

Gene or region	Clade	A	B	C	D
COI	A	**0-7.05**			
	B	8.11-13.93	**0-2.12**		
	C	10.58-15.52	8.99-10.05	**0-0.35**	
	D	11.99-16.93	11.99-13.23	11.29-11.99	**0.35-0.53**
16S rDNA	A	**0-1.84**			
	B	3.16-4.49	**0-1.30**		
	C	6.87-7.69	6.61-7.67	**0-0.79**	
	D	8.73-10.05	8.17-9.23	9.76-10.05	**0.00**
Combined mtDNA	A	**0-4.86**			
	B	6.34-11.42	**0-4.43**		
	C	9.31-12.16	8.45-10.38	**0-0.42**	
	D	10.99-13.74	7.58-11.51	10.78-11.21	**0.21-0.31**
ITS1	A	**0-0.66**			
	B	2.38-3.22	**0-1.53**		
	C	4.35-4.71	4.79-5.28	**0.00**	
	D	6.91-8.66	6.45-8.01	7.62-9.01	**0-1.35**
