# Supplementary material for: Population genetic structure of Indoplanorbis exustus (Gastropoda: Planorbidae) in Thailand and its infection with trematode cercariae
Source: PLoS One. 2024 Jan 26;19(1):e0297761. doi: 10.1371/journal.pone.0297761 (PMC10817173; doi:10.1371/journal.pone.0297761)
Supplement: S6 Table — (PDF) [file pone.0297761.s009.pdf]

S6 Table. Population pairwise F_{ST} between 16 populations of *I. exustus* based on mitochondrial cytochrome c oxidase I gene sequences.

Populations	Lamphun	Chaiyaphum	Khon Kaen	Udon Thani	Phitsanulok	Sukhothai	Phichit	Chai Nat	Sing Buri	Nakhon Sawan	Ang Thong	Nakhon Nayok	Tak	Chon Buri	Pattani	Songkhla
Lamphun	0.000															
Chaiyaphum	-0.006	0.000														
Khon Kaen	-0.304	-0.317	0.000													
Udon Thani	0.000	-0.030	-0.304	0.000												
Phitsanulok	-0.021	0.011	-0.303	-0.052	0.000											
Sukhothai	-0.153	-0.174	0.000	-0.153	-0.174	0.000										
Phichit	0.081	0.034	-0.325	0.025	-0.018	-0.187	0.000									
Chai Nat	0.018	0.024	-0.321	-0.067	-0.002	-0.187	-0.016	0.000								
Sing Buri	-0.086	0.060	-0.215	0.022	0.064*	-0.096	0.076*	0.087*	0.000							
Nakhon Sawan	0.052	0.012	-0.321	0.012	-0.025	-0.180	0.002	-0.026	0.059	0.000						
Ang Thong	-0.304	-0.317	0.000	-0.304	-0.303	0.000	-0.325	-0.321	-0.215	-0.321	0.000					
Nakhon Nayok	-0.304	-0.317	0.000	-0.304	-0.303	0.000	-0.325	-0.321	-0.215	-0.321	0.000	0.000				
Tak	0.166	0.083	0.000	0.166	-0.001	0.000	0.029	-0.001	0.109*	0.060	0.000	0.000	0.000			
Chon Buri	0.027	0.013	-0.323	-0.085	-0.008	-0.184	0.006	-0.024	0.066*	-0.006	-0.323	-0.323	0.042	0.000		
Pattani	-0.074	-0.106	-0.200	0.068	-0.113	0.000	0.195	-0.076	-0.047	0.148	-0.200	-0.200	0.531	-0.022	0.000	
Songkhla	0.136	0.084	-0.270	-0.114	0.011	-0.143	0.031	-0.001	0.118*	0.032	-0.270	-0.270	0.052	-0.008	0.210	0.000

Asterisks (*) indicate statistical significance of $P < 0.05$.
