# Supplementary material for: Population genetic structure of Indoplanorbis exustus (Gastropoda: Planorbidae) in Thailand and its infection with trematode cercariae
Source: PLoS One. 2024 Jan 26;19(1):e0297761. doi: 10.1371/journal.pone.0297761 (PMC10817173; doi:10.1371/journal.pone.0297761)
Supplement: S8 Table — (PDF) [file pone.0297761.s011.pdf]

S8 Table. Population pairwise F_{ST} between 16 populations of *I. exustus* of the combined mtDNA sequences.

Populations	Lamphun	Chaiyaphum	Khon Kaen	Udon Thani	Phitsanulok	Sukhothai	Phichit	Chai Nat	Sing Buri	Nakhon Sawan	Ang Thong	Nakhon Nayok	Tak	Chon Buri	Pattani	Songkhla
Lamphun	0.000															
Chaiyaphum	-0.006	0.000														
Khon Kaen	-0.304	-0.317	0.000													
Udon Thani	0.000	-0.030	-0.304	0.000												
Phitsanulok	-0.021	0.011	-0.303	-0.052	0.000											
Sukhothai	-0.153	-0.174	0.000	-0.153	-0.174	0.000										
Phichit	0.081	0.034	-0.325	0.025	-0.018	-0.187	0.000									
Chai Nat	0.018	0.024	-0.321	-0.067	-0.002	-0.187	-0.016	0.000								
Sing Buri	-0.086	0.057	-0.220	0.018	0.063*	-0.100	0.072	0.085*	0.000							
Nakhon Sawan	0.052	0.012	-0.321	0.012	-0.025	-0.180	0.002	-0.026	0.055	0.000						
Ang Thong	-0.090	-0.144	0.000	0.225	-0.149	0.250	0.416	-0.055	-0.097	0.352	0.000					
Nakhon Nayok	-0.304	-0.317	0.000	-0.304	-0.303	0.000	-0.325	-0.321	-0.220	-0.321	0.000	0.000				
Tak	0.166	0.083	0.000	0.166	-0.001	0.000	0.029	-0.001	0.105*	0.060	0.776	0.000	0.000			
Chon Buri	0.027	0.013	-0.323	-0.085	-0.008	-0.184	0.006	-0.024	0.062*	-0.006	0.050	-0.323	0.042	0.000		
Pattani	-0.074	-0.106	-0.200	0.068	-0.113	0.000	0.195	-0.076	-0.053	0.148	0.045	-0.200	0.531	-0.022	0.000	
Songkhla	0.214*	0.180*	0.051	0.187*	0.111*	0.135	0.277*	0.164*	0.184*	0.257*	0.262*	0.051	0.344*	0.198*	0.213	0.000

Asterisks (*) indicate statistical significance of $P < 0.05$.
