# Supplementary material for: Population genetic structure of Indoplanorbis exustus (Gastropoda: Planorbidae) in Thailand and its infection with trematode cercariae
Source: PLoS One. 2024 Jan 26;19(1):e0297761. doi: 10.1371/journal.pone.0297761 (PMC10817173; doi:10.1371/journal.pone.0297761)
Supplement: S9 Table — (PDF) [file pone.0297761.s012.pdf]

## S9 Table. Population pairwise $F_{ST}$ between 16 populations of *I. exustus* based on ITS1 sequences.

| Populations | Lamphun | Chaiyaphum | Khon Kaen | Udon Thani | Phitsanulok | Sukhothai | Phichit | Chai Nat | Sing Buri | Nakhon Sawan | Ang Thong | Nakhon Nayok | Tak | Chon Buri | Pattani | Songkhla |
|---|---|---|---|---|---|---|---|---|---|---|---|---|---|---|---|---|
| Lamphun | 0.000 | | | | | | | | | | | | | | | |
| Chaiyaphum | -0.008 | 0.000 | | | | | | | | | | | | | | |
| Khon Kaen | -0.304 | -0.317 | 0.000 | | | | | | | | | | | | | |
| Udon Thani | 0.000 | -0.040 | 0.000 | 0.000 | | | | | | | | | | | | |
| Phitsanulok | 0.023 | -0.013 | -0.315 | -0.074 | 0.000 | | | | | | | | | | | |
| Sukhothai | 0.802* | 0.707* | 1.000 | 1.000* | 0.779* | 0.000 | | | | | | | | | | |
| Phichit | 0.108 | 0.041 | 0.000 | 0.000 | -0.027 | 1.000* | 0.000 | | | | | | | | | |
| Chai Nat | 0.203 | 0.110 | 0.000 | 0.000 | -0.002 | 1.000* | 0.000 | 0.000 | | | | | | | | |
| Sing Buri | 0.050 | 0.050 | -0.265 | -0.025 | 0.006 | 0.778* | 0.033 | 0.077 | 0.000 | | | | | | | |
| Nakhon Sawan | -0.008 | 0.001 | -0.321 | -0.051 | 0.029 | 0.726* | 0.023 | 0.082 | 0.042 | 0.000 | | | | | | |
| Ang Thong | -0.304 | -0.317 | 0.000 | 0.000 | -0.315 | 1.000 | 0.000 | 0.000 | -0.265 | -0.321 | 0.000 | | | | | |
| Nakhon Nayok | 0.346 | 0.315 | 0.000 | 0.538 | 0.558* | 0.647 | 0.710 | 0.808* | 0.549* | 0.352 | 0.000 | 0.000 | | | | |
| Tak | 0.053 | 0.042 | -0.329 | -0.076 | 0.001 | 0.893* | -0.022 | 0.012 | 0.042 | -0.031 | -0.329 | 0.625 | 0.000 | | | |
| Chon Buri | 0.090 | 0.029 | 0.000 | 0.000 | -0.033 | 1.000* | 0.000 | 0.000 | 0.025 | 0.012 | 0.000 | 0.687 | -0.029 | 0.000 | | |
| Pattani | 0.460 | 0.396 | 0.368 | 0.675* | 0.571* | 0.800 | 0.791 | 0.859* | 0.586* | 0.424* | 0.368 | -0.200 | 0.671 | 0.775* | 0.000 | |
| Songkhla | 0.191 | 0.101 | 0.000 | 0.000 | -0.005 | 1.000* | 0.000 | 0.000 | 0.072 | 0.075* | 0.000 | 0.798 | 0.008 | 0.000 | 0.853* | 0.000 |

Asterisks (*) indicate statistical significance of $P < 0.05$.
